# Determination of the physiological and pathological roles of E2F3 in adult tissues

**DOI:** 10.1038/s41598-017-09494-6

**Published:** 2017-08-30

**Authors:** Ivonne Gamper, Deborah L. Burkhart, Megan J. Bywater, Daniel Garcia, Catherine H. Wilson, Peter A. Kreuzaler, Mark J. Arends, Yao-Wu Zheng, Alessandra Perfetto, Trevor D. Littlewood, Gerard I. Evan

**Affiliations:** 1 Department of Biochemistry, University of Cambridge, Cambridge, UK; 2 The Salk Institute for Biological Sciences, 10010 North Torrey Pines Rd, La Jolla, CA 92037 USA; 3 Pathology Department, University of Cambridge, Cambridge, UK; 4 Cardiovasular Research Institute, Department of Medicine, University of California, San Francisco, San Francisco, CA 94158 USA; 5 Present Address: Division of Pathology, Centre for Comparative Pathology, University of Edinburgh, Cancer Research UK Edinburgh Centre, Institute of Genetics and Molecular Medicine, Crewe Road, Edinburgh, UK; 6 Present Address: Transgenic Research Center, School of Life Sciences, Northeast Normal University, Changchun, China

## Abstract

While genetically engineered mice have made an enormous contribution towards the elucidation of human disease, it has hitherto not been possible to tune up or down the level of expression of any endogenous gene. Here we describe compound genetically modified mice in which expression of the endogenous *E2f3* gene may be either reversibly elevated or repressed in adult animals by oral administration of tetracycline. This technology is, in principle, applicable to any endogenous gene, allowing direct determination of both elevated and reduced gene expression in physiological and pathological processes. Applying this switchable technology to the key cell cycle transcription factor E2F3, we demonstrate that elevated levels of E2F3 drive ectopic proliferation in multiple tissues. By contrast, E2F3 repression has minimal impact on tissue proliferation or homeostasis in the majority of contexts due to redundancy of adult function with E2F1 and E2F2. In the absence of E2F1 and E2F2, however, repression of E2F3 elicits profound reduction of proliferation in the hematopoietic compartments that is rapidly lethal in adult animals.

## Introduction

E2F3 is one of an eight-member family of transcription factors that are critical for cell cycle progression and differentiation and are the principal targets of the RB tumour suppressor and its p107 and p130 siblings (reviewed in ref. 1). Within the E2F family, E2F1-3 are considered “activator E2Fs” whose principal function, once unleashed by hyperphosphorylation of RB in response to mitogenic signalling, is to engage expression of genes necessary for the G0-S cell cycle transition.

The three activator E2F transcription factors are believed to be broadly interchangeable and functionally redundant^1–3^ although various additional unique and often subtle properties have been attributed to each individual member. For example, germ-line and conditional, Cre-mediated deletions of *E2f3 in vivo* intimate that E2F3 has unique roles not shared with other activator E2F family members^4, 5^ during embryonic development^6, 7^, in myogenic differentiation^8^, neuronal migration^9^, DNA damage responses^10^ and in some cancers such as HER2-driven mammary tumours^11, 12^, Ewing’s sarcoma and prostate cancer^13^. Moreover, whereas *E2f1* ^−/−^, *E2f2* ^−/−^, and *E2f1* ^−/−^; *E2f2* ^−/−^ double knockout mice are all viable, exhibiting only subtle tumourigenic and developmental deficits^1^, deletion of *E2f3* elicits profound embryonic lethality, principally due to dysfunctional cardiac development^6^. Such embryonic lethality, complete in a pure *129/Sv* background, but still 75% penetrant in a mixed (*129/Sv* × *C57BL/6*) background, greatly complicates any genetic study of E2F3 function in adult tissues. Nonetheless, much of the biological output of E2F3 appears redundant with that of E2F1 and E2F2, and overall the most uniform detected phenotype of germ-line *E2f3* deletion is partial reduction in proliferative activity in cultured cells^14, 15^.

All germ-line and conditional *E2f3* knock-out studies are complicated by the likelihood of developmental adaptation and compensatory rewiring during embryonic and tissue development and, in the case of conditional knock-outs, by incomplete deletion of the conditional allele, generating chimeric target tissues. Knock-out strategies also suffer from the inherent limitation of their irreversibility, precluding investigation of the impact of, say, transient E2F3 ablation on normal or neoplastic tissue maintenance.

The other principal investigative strategy for studying the *in vivo* function of a protein is to express it ectopically in transgenic mice. In the case of E2F3, all such studies, whether *keratin 5* promoter-driven in epidermis^16^, *pro-opiomelanocortin*-driven in pituitary^17^ or *alphaA-crystallin* promoter-driven in lens fibre cells^18^, concur that precocious expression of E2F3 elicits ectopic proliferation. Elevated E2F3 expression also increases apoptosis in epidermis and lens fibre cells^16, 18^. Unfortunately, such classical transgenic mice also suffer from inherent shortcomings: they are prone to variegated patterns and levels of transgene expression and to sporadic inactivation/erosion of transgene expression due to promoter methylation and gene silencing. Furthermore, their phenotypes ultimately depend as much on the biology of the specific promoter used to drive the *E2f3* transgene as on E2F3 itself. Hence, while the collective data from the existing *E2f3* knock-out and transgenic models all support the notion that *E2f3* expression is critically involved in cell proliferation, development and cancer, the precise, real-time role of *E2f3* in adult tissues *in vivo* remains unclear and is difficult to address with current experimental systems.

To circumvent the above issues, we generated a novel switchable gene expression mouse model that allows for tuneable expression (either elevated or repressed) of the endogenous *E2f3* gene in adult mice. Importantly, in this model *E2f3* is expressed normally during development, mitigating adaptive compensation for E2F3 loss during development but allowing E2F3 function in adults to be acutely and reversibly switched up or down, either systemically or tissue-by-tissue. Using this novel mouse, we directly determine the role of E2F3 in maintaining normal adult tissue architecture. Further, by combining our switchable *E2f3* mouse with existing *E2f1* and *E2f2* knockout animals, we constrain all classically mitogenic E2F1-3 activity to flow through our tuneable *E2f3* allele. This allows us to address the impact of acute, transient repression of combined E2F1-3 function on normal tissues, thereby exploring the potential existence of a temporal therapeutic window for inhibition of mitogenic E2Fs.

## Results

**Figure 1:**
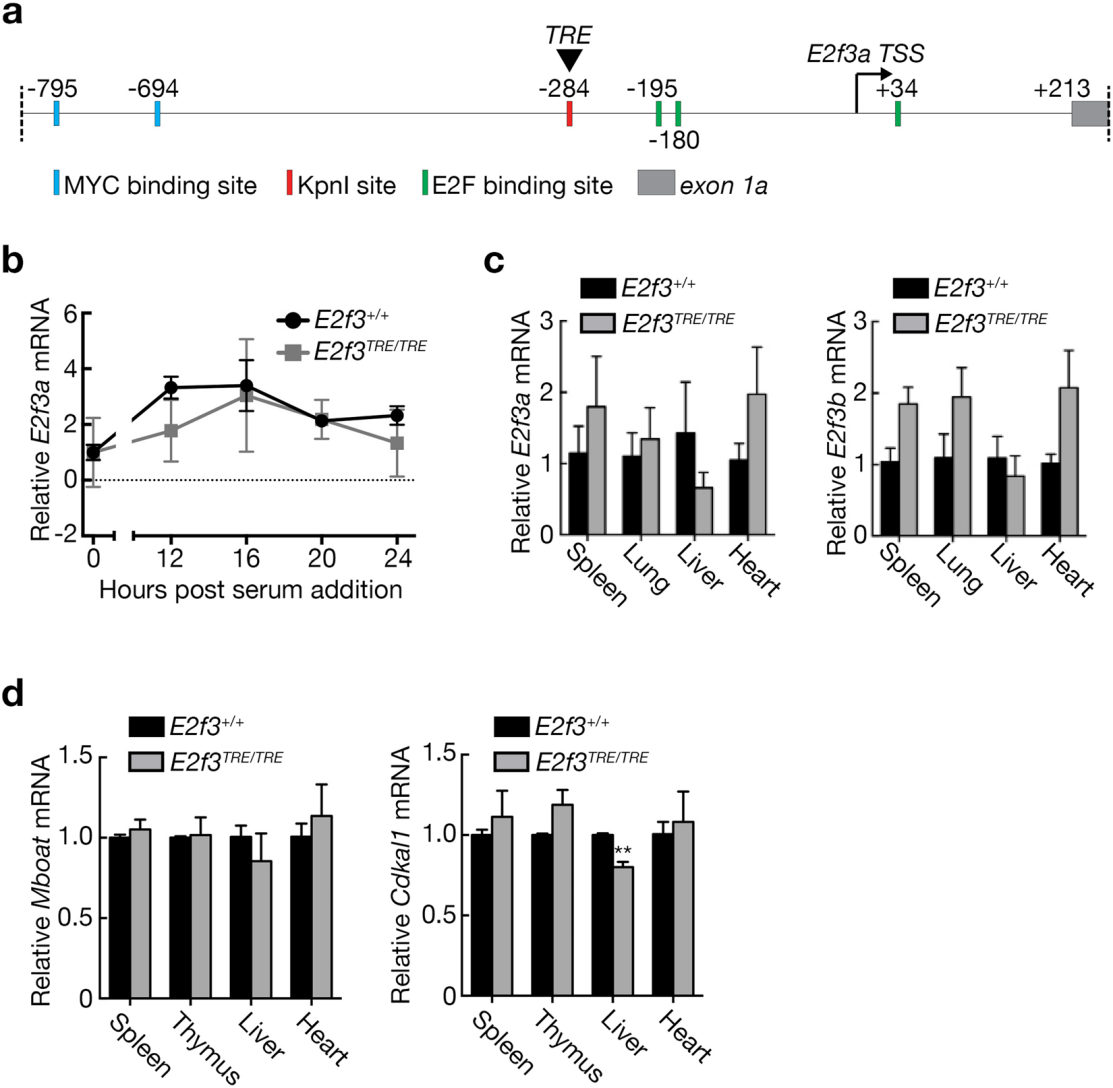
TRE insertion into the *E2f3* promoter does not affect normal *E2f3* regulation. (**a**) Schematic of *E2f3* promoter with relative positions of known transcription factor binding sites, the transcription start site, and the TRE insertion site. Blue box- Myc binding site; green box- E2F binding site; grey box- E2f3a exon 1a; arrow- transcription start site; red box-TRE insertion site. (**b**) Quantitative RT-PCR analysis of *E2f3a* expression in quiescent wild type and *E2f3* ^*TRE/TRE*^ MEFs following serum stimulation. *E2f3a* expression is normalized to *HPRT* and data normalized to the quiescent sample within each genotype. *n* = 3 independent MEF lines; error bars, s.d. (**c**) Quantitative RT-PCR analysis of *E2f3a* and *E2f3b* mRNA expression in adult wild type and *E2f3* ^*TRE/TRE*^ mouse tissues. Expression is normalized to *HPRT* and relative to the mean of the wild type tissues. *n* = 3 mice; error bars, s.e.m. (**d**) Quantitative RT-PCR analysis of *Mboat1* and *Cdkal1* expression in adult wild type and *E2f3* ^*TRE/TRE*^ mouse tissues. Expression is normalized to *actin* and relative to the mean of the wild type samples. *n* = 3 mice; error bars, s.e.m. Two-tailed t-tests; *Cdkal1*: liver, **P = 0.004.

To generate mice in which the endogenous *E2f3* gene may be reversibly switched on or off at will, a heptameric tetracycline-response element (TRE) derived from the pTRE2 vector (Clontech) was targeted via homologous recombination to the promoter of the endogenous *E2f3* locus in mouse embryonic stem cells (mESCs), from which *E2f3* ^*TRE*^ mice were derived. The TRE was placed in close proximity to the *E2f3a* promoter yet avoiding the critical Myc and E2F binding sites described previously^19^ (Fig. 1a). Insertion of this TRE then allows for ectopic control of *E2f3* expression by serving as a binding site for tetracycline-regulatable transcriptional activators or repressors. Accordingly, *E2f3* ^*TRE*^ mice were then crossed into mice ubiquitously expressing the reverse tetracycline-dependent transactivator (rtTA), for reversible enforced endogenous *E2f3* induction, or into mice ubiquitously expressing either the tTS(kid) tetracycline regulated transcriptional repressor or the reverse tetracycline-controlled transcriptional silencer (rtTS), both potentially enabling reversible repression of endogenous *E2f3*.

### TRE insertion into the *E2f3* promoter does not perturb normal *E2f3* regulation

The great majority of *E2f3* knock-out mice die between E13.5 and P2 dpc^20^, exhibiting fatal cardiac abnormalities and typical signs of congestive heart failure. By contrast, homozygous *E2f3* ^*TRE/TRE*^ mice were born at expected Mendelian ratios, indicating that TRE insertion does not critically interfere with control of *E2f3* expression during development. In normal somatic cells, the kinetics and levels of *E2f3* expression, specifically the *E2f3a* isoform, are tightly regulated in a cell cycle-dependent manner by multiple transcription factors, including Sp1, E2F and Myc families^19^. To assess any impact of the TRE insertion on normal *E2f3* cell-cycle expression, quiescent wild-type and *E2f3* ^*TRE/TRE*^ MEFs were serum-stimulated and *E2f3a* expression monitored over time. Kinetics and levels of *E2f3a* expression were essentially identical in *E2f3* ^*TRE/TRE*^ and wild-type MEFs, in both cases peaking at 16 hours post serum-stimulation (Fig. 1b). We similarly saw no statistically significant differences in either *E2f3a* or *E2f3b* mRNA expression in adult *E2f3* ^*TRE/TRE*^ versus wild-type mouse spleen, lung, liver, or heart (Fig. 1c). Insertion of the TRE into the *E2f3* promoter also had no discernible impact on the expression of genes located to either side of *E2f3* on mouse chromosome 13 - *Mboat1* (50 kb telomeric) and *Cdkal1* (151 kb centromeric) - in the tissues examined, save for a modest decrease in the liver (Fig. 1d). Taken together, these data indicate that insertion of the 300 bp TRE into the *E2f3* promoter has negligible impact on normal expression and regulation of either *E2f3* or its flanking genes.

### Reversible induction of endogenous *E2f3* in *E2f3*^*TRE/TRE*^*mice*

**Figure 2:**
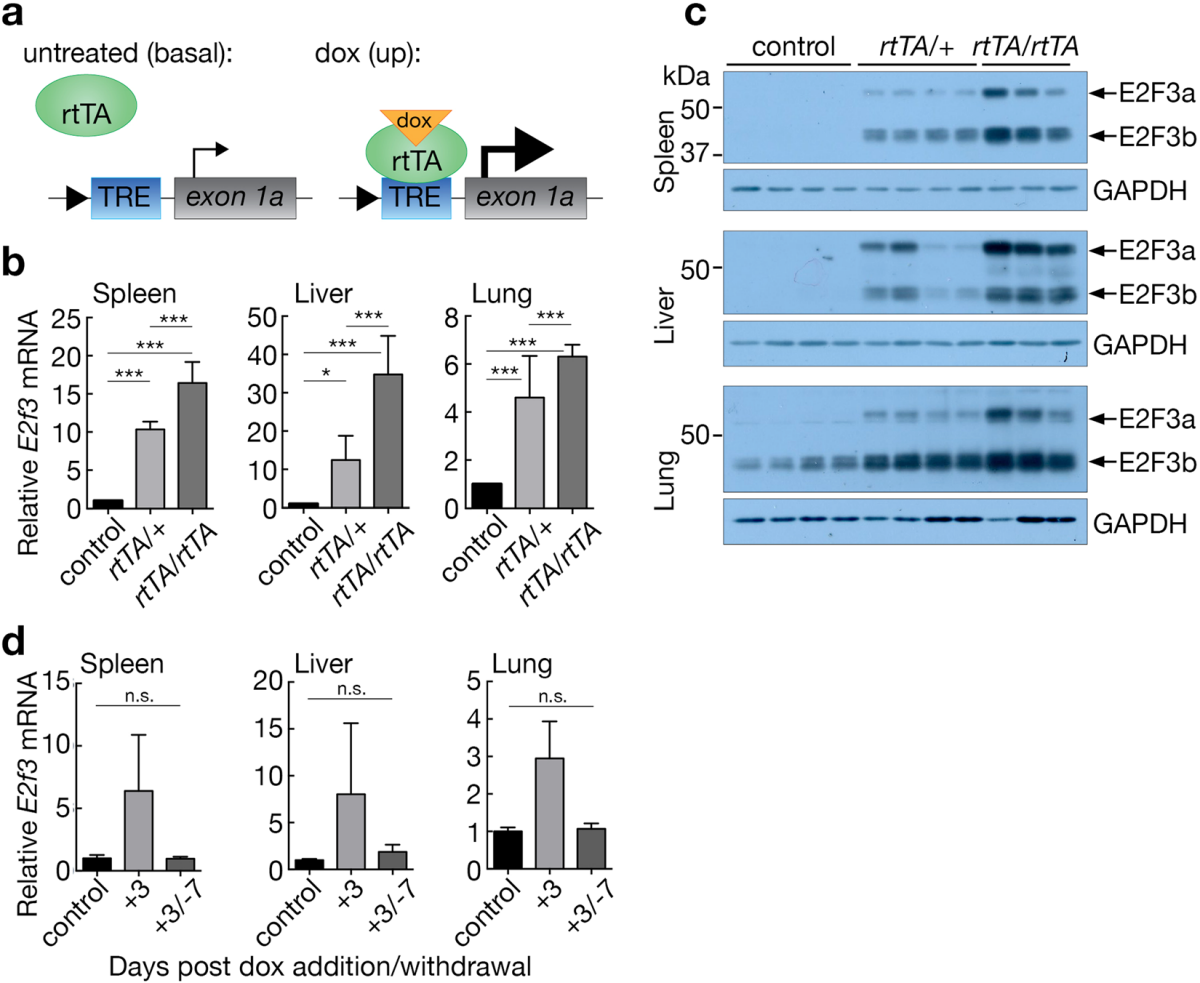
*E2f3* expression may be reversibly induced in *E2f3* ^*TRE/TRE*^ mice. (**a**) Schematic of rtTA driven expression from the *E2f3* ^*TRE*^ locus. Grey box- exon, blue box- TRE element, green oval- reverse tetracycline trans activator, orange triangle- doxycycline. (**b**) Quantitative RT-PCR analysis of *E2f3* expression in adult control (*n* = 6(spleen, lung), n = 5(liver)), *E2f3* ^*TRE/TRE*^; *Rosa26* ^*rtTA/*+^ (*n* = 4) and *E2f3* ^*TRE/TRE*^; *Rosa26* ^*rtTA/rtTA*^ (*n* = 3) mouse tissues following administration of doxycycline for three days. Expression is normalized to *HPRT* and relative to the mean of the control samples. Error bars, s.e.m. Two-way ANOVA with a Tukey’s multiple comparisons test; *P < 0.5, ***P < 0.001. (**c**) Immunoblot analysis of E2F3 protein levels in adult control, *E2f3* ^*TRE/TRE*^; *Rosa26* ^*rtTA/*+^ and *E2f3* ^*TRE/TRE*^ *Rosa26* ^*rtTA/rtTA*^ mouse tissues following administration of doxycycline for three days. Replicate samples are generated from tissues isolated from independent mice. Expression of GAPDH is included as a loading control. Refer to Supplementary Fig. 11 for full images. (**d**) Quantitative RT-PCR analysis of *E2f3* expression in adult control (*n* = 3) and *E2f3* ^*TRE/TRE*^; *Rosa26* ^*rtTA/*+^ tissues isolated from mice following administration of doxycycline for three days only (+3, *n* = 2) or for three days with subsequent withdrawal for seven days (+3/−7, *n* = 5). Expression is normalized to *HPRT* and relative to the mean of the control samples. Error bars, s.e.m. Two-tailed t-test: control vs. +3/−7, *P* = 0.82 (spleen), 0.10 (liver), 0.52 (lung); n.s., not significant. Control genotypes include *E2f3* ^*TRE/TRE*^ in the presence and absence of doxycycline and *E2f3* ^*TRE/TRE*^; *Rosa26* ^*rtTA/*+^ and *E2f3* ^*TRE/TRE*^ *Rosa26* ^*rtTA/rtTA*^ maintained in the absence of doxycycline.

To induce *E2f3* ectopically from the endogenous *E2f3* ^*TRE*^ allele, we crossed *E2f3* ^*TRE/TRE*^ mice into the *Rosa26* ^rtTA*M2^ background^21^, which ubiquitously expresses the reverse tetracycline-dependent transactivator (rtTA) driven from either one (*Rosa26* ^*rtTA/*+^) or two alleles (*Rosa26* ^*rtTA/rtTA*^) of the pan-active *Rosa26* promoter (Fig. 2a). In the absence of doxycycline, both *E2f3* ^*TRE/TRE*^; *Rosa26* ^*rtTA/*+^ and *E2f3* ^*TRE/TRE*^; *Rosa26* ^*rtTA/rtTA*^ mice were born at normal Mendelian ratios and appeared indistinguishable from their *rtTA*-negative control littermates (not shown). Administration of doxycycline to either *E2f3* ^*TRE/TRE*^; *Rosa26* ^*rtTA/*+^ or *E2f3* ^*TRE/TRE*^; *Rosa26* ^*rtTA/rtTA*^ adult mice significantly increased *E2f3* expression in all tested tissues within 3 days (Fig. 2b,c). Moreover, mice homozygous for the *rtTA* allele consistently expressed 1.4 to 2.8 fold higher levels of *E2f3* mRNA in their tissues relative to mice with a single *rtTA* allele. Hence, the extent of *E2f3* over-expression in *E2f3* ^*TRE/TRE*^; *Rosa26* ^*rtTA*^ mice is proportional to the level of rtTA expressed. Tissues of doxycycline-treated *E2f3* ^*TRE/TRE*^; *Rosa26* ^*rtTA/*+^ and *E2f3* ^*TRE/TRE*^; *Rosa26* ^*rtTA/rtTA*^ mice over-expressed both E2F3a and E2F3b protein isoforms (Fig. 2c). By contrast, expression of the two genes flanking *E2f3*, *Mboat* and *Cdkal1*, was unaffected by expression of rtTA combined with doxycycline administration (Supplementary Fig. 1). Induced over-expression of *E2f3* was rapidly reversed upon withdrawal of doxycycline (Fig. 2d).

### Reversible repression of endogenous *E2f3* expression in *E2f3*^*TRE/TRE*^*mice*

**Figure 3:**
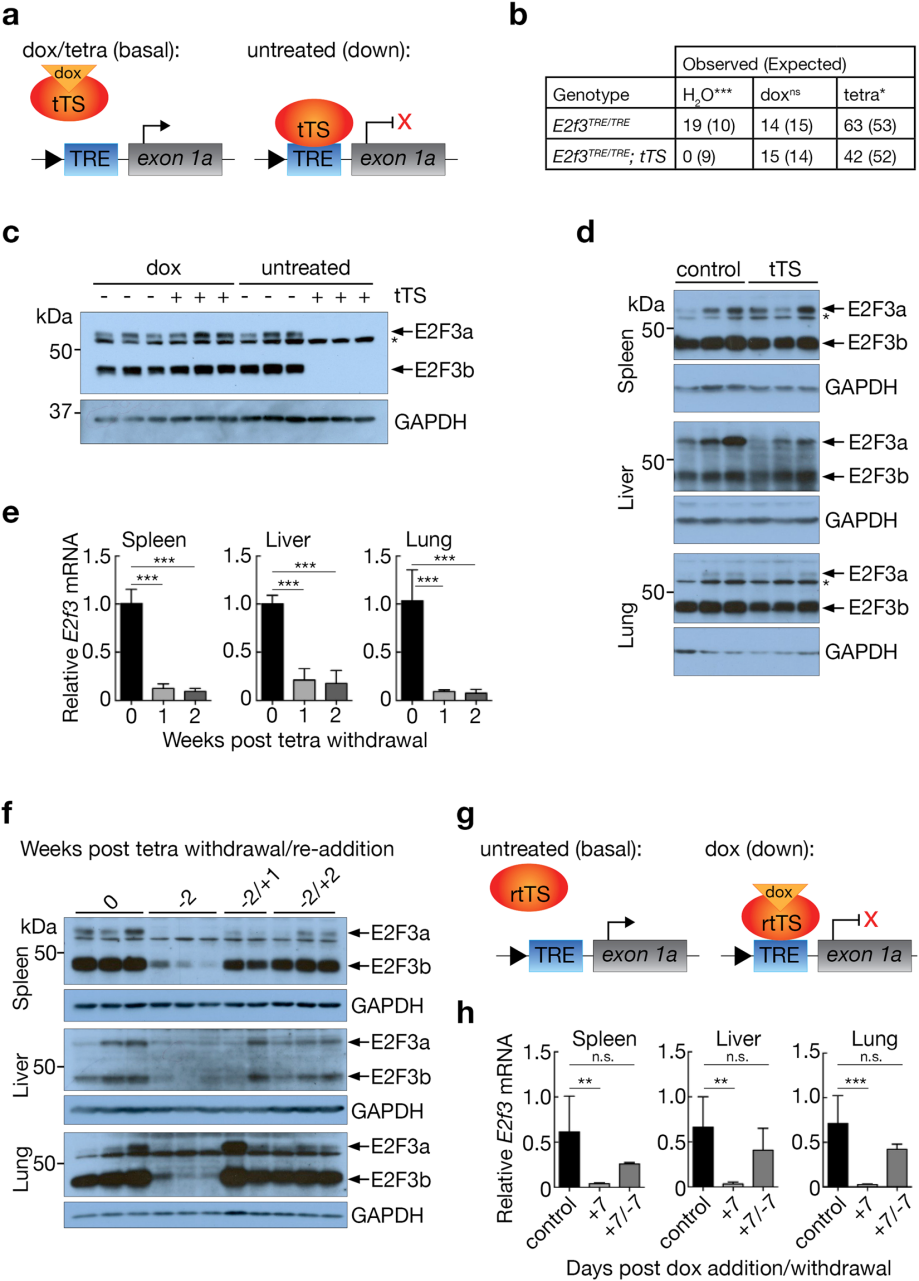
*E2f3* expression may be reversibly repressed in *E2f3* ^*TRE/TRE*^ mice. (**a**) Schematic of tTS(kid)-driven repression. Grey box- exon, blue box- TRE element, red oval- tetracycline regulated repressor, orange triangle- doxycycline. (**b**) Genotypes of live births from *E2f3* ^*TRE/TRE*^; *β-actin-tTS* crosses maintained on normal drinking water (H_2_O), with doxycycline (dox), or with tetracycline (tetra). Chi-square test, ***P < 0.0001 (water), 0.85(dox), 0.04*(tetra). ns = not significant. (**c**) Immunoblot analysis of E2F3 protein levels in *E2f3* ^*TRE/TRE*^ (−) and *E2f3* ^*TRE/TRE*^; *β-actin-tTS* (+) cultured mouse embryonic fibroblasts, developed and maintained either in the presence (dox) or absence (untreated) of doxycycline. Replicate samples represent independently isolated embryo cultures. GAPDH is included as a loading control. Refer to Supplementary Fig. 12 for full images. (**d**) Immunoblot analysis of E2F3 protein levels in adult control *E2f3* ^*TRE/TRE*^ and *E2f3* ^*TRE/TRE*^; *β-actin-tTS* tissues isolated from mice maintained on tetracycline. Replicate samples are generated from tissues isolated from independent mice. GAPDH is included as a loading control, *non-specific band. Refer to Supplementary Fig. 13 for full images. (**e**) Quantitative RT-PCR analysis of *E2f3* expression in *E2f3* ^*TRE/TRE*^; *β-actin-tTS* mice maintained on tetracycline (*n* = 3) or following 1 (*n* = 4) or 2 (*n* = 3) weeks withdrawal of tetracycline. Expression is normalized to *HPRT* and relative to the mean of the samples maintained on tetracycline. Error bars, s.e.m. One-way ANOVA with a Tukey’s multiple comparisons test, ***P < 0.001. (**f**) Immunoblot analysis of E2F3 protein levels in *E2f3* ^*TRE/TRE*^; *β-actin-tTS* mice maintained on tetracycline throughout (0), removed from tetracycline for 2 weeks (−2), or removed from tetracycline for 2 weeks followed by re-addition of tetracycline for 1 week (−2/+1) or 2 weeks (−2/+2). Replicate samples are generated from tissues isolated from independent mice. GAPDH is included as a loading control. Refer to Supplementary Fig. 14 for full images. (**g**) Schematic of rtTS driven repression. Grey box- exon, blue box- TRE element, red oval- reverse tetracycline trans silencer, orange triangle- doxycycline. (**h**) Quantitative RT-PCR analysis of *E2f3* in control *E2f3* ^*TRE/TRE*^ (*n* = 12 (spleen, liver), 11 (lung)) and *E2f3* ^*TRE/TRE*^; *Rosa26* ^*CAGrtTS/*+^ mouse tissues in the presence of 2 g/L doxycycline for seven days ( + 7, *n* = 5) or for seven days and a subsequent seven days post withdrawal ( + 7/−7, *n* = 4). Expression is normalized to *HPRT* and relative to the mean of the control samples. Error bars, s.e.m. One-way ANOVA with a Tukey’s multiple comparisons test, **P < 0.01, ***P < 0.001, n.s. = not significant.

We used two complementary approaches to repress *E2f3* expression. In the first, we crossed *E2f3* ^*TRE/TRE*^ mice to a transgenic strain that expresses the tetracycline regulated repressor, tTS^22^ driven from the broadly active *β-actin* promoter, as described previously^23^. tTS is a fusion protein combining the bacterial tet repressor (TetR) with the KRAB-AB domain of the Kid-1 transcriptional repressor. In the absence of antibiotic (tetracycline or doxycycline), tTS binds to its cognate TRE-element, whereupon it represses transcription of proximal genes (Fig. 3a). *E2f3* ^*TRE/TRE*^; *β-actin-tTS* pups were born at normal Mendelian frequency from mothers administered with 100 mg/L doxycycline throughout pregnancy to inactivate the tTS repressor (Fig. 3b). Furthermore normal levels of both E2F3a and b proteins were expressed in MEFs derived from embryos developed in the continuous presence of doxycycline (Fig. 3c). By contrast, neither E2F3a nor E2F3b proteins was detectable in MEFs derived from embryos continuously deprived of doxycycline throughout development and cell culture (Fig. 3c). Moreover, no *E2f3* ^*TRE/TRE*^; *β-actin-tTS* pups were born in the absence of doxycycline (Fig. 3b) and anatomical analysis of antibiotic-deprived *E2f3* ^*TRE/TRE*^; *β-actin-tTS* embryos collected at E11.5 indicated the same severe reduction in heart trabeculation^6^ (Supplementary Fig. 2) as that reported for germ-line *E2f3* knock-out mice^15, 20^. Hence, *E2f3* ^*TRE/TRE*^; *β-actin-tTS* embryos in the absence of doxycycline accurately replicate the developmental phenotype of germ-line *E2f3* knockout mice. Hence, doxycycline efficiently keeps the tTS repressor inactive, permitting normal developmental *E2f3* expression.

Doxycycline, like other members of the tetracycline family of antibiotics, accumulates in developing bones^24^. Such accumulation serves as an antibiotic reservoir that delays its clearance after it is withdrawn from *E2f3* ^*TRE/TRE*^; *β-actin-tTS* pups born to mothers previously treated with doxycycline throughout gestation and weaning (unpublished observations and^25, 26^). To circumvent the impact of this delay on switchably repressing *E2f3* expression in *E2f3* ^*TRE/TRE*^ mice, we replaced doxycycline with tetracycline, which is less lipophilic and has a shorter biological half-life^27^. Maintenance of pregnant mothers on drinking water containing tetracycline concentration at 50 mg/L proved sufficient to maintain normal embryonic development of *E2f3* ^*TRE/TRE*^; *β-actin-tTS* pups (Fig. 3b) and culminated in neonate and adult mice indistinguishable from their *E2f3* wild-type littermates. This effective level of tetracycline was then used for all subsequent studies unless otherwise noted.

We next assessed the efficacy and reversibility of *E2f3* repression in mouse tissues *in vivo* using the tTS system. We first confirmed that administration of tetracycline throughout development maintains expression of *E2f3* at ostensibly normal levels in all tested adult tissues (Fig. 3d). However, withdrawal of tetracycline from adult mice hitherto maintained on tetracycline induced ubiquitous and profound repression of *E2f3*, evident within one week and thereafter sustained (Fig. 3e), with concomitant reduction of both E2F3a and E2F3b proteins (Fig. 3f). Expression of *E2f3*’s two neighbouring genes, *Mboat1* and *Cdkal1*, was unaffected except for a modest decrease in *Cdkal1* in spleen (Supplementary Fig. 3). Subsequent restoration of tetracycline to the drinking water of *E2f3*-repressed *E2f3* ^*TRE/TRE*^; *β-actin-tTS* mice rapidly restored normal E2f3 expression (Fig. 3f).

We also reconfigured the tTS repression system from one where tetracycline inactivates the repressor to one where the antibiotic activates it. Such a configuration has clear advantages in situations requiring long periods of normal *E2f3* expression punctuated by acute periods of *E2f3* repression. To do this, we generated a novel *Rosa26* ^*CAG-rtTS*^ strain that ubiquitously expresses a chimeric protein formed from the fusion of the reverse tetracycline regulated region of rtTA^28^ with the robust transcriptional silencer derived from the KRAB domain of Kid-1 protein^22, 29, 30^, similar to the one previously described^31^. The resulting rtTS repressor is dependent on tetracycline or doxycycline for its repressing activity (Fig. 3g). Administration of doxycycline to *E2f3* ^*TRE/TRE*^; *Rosa26* ^*CAG-rtTS/*+^ mice for one week repressed *E2f3* transcription to nearly undetectable levels in all tissues tested; this repression was reversible upon withdrawal of doxycycline (Fig. 3h). Together these two systems, tTS and rtTS, allow for unprecedentedly rapid and flexible switching of target gene activity in adult tissues *in vivo*.

### Consequences of differential expression of E2F3 in adult tissues

Because switchable genetic systems obviate the complications of both embryonic lethality and adaptive compensation induced by absence or constitutive over-expression of genes during development, we could uniquely use our switchable *E2f3* ^*TRE/TRE*^ mice specifically to determine the biological functions of E2F3 in adult tissues in mice that had developed in the presence of normal E2F3 expression.

**Figure 4:**
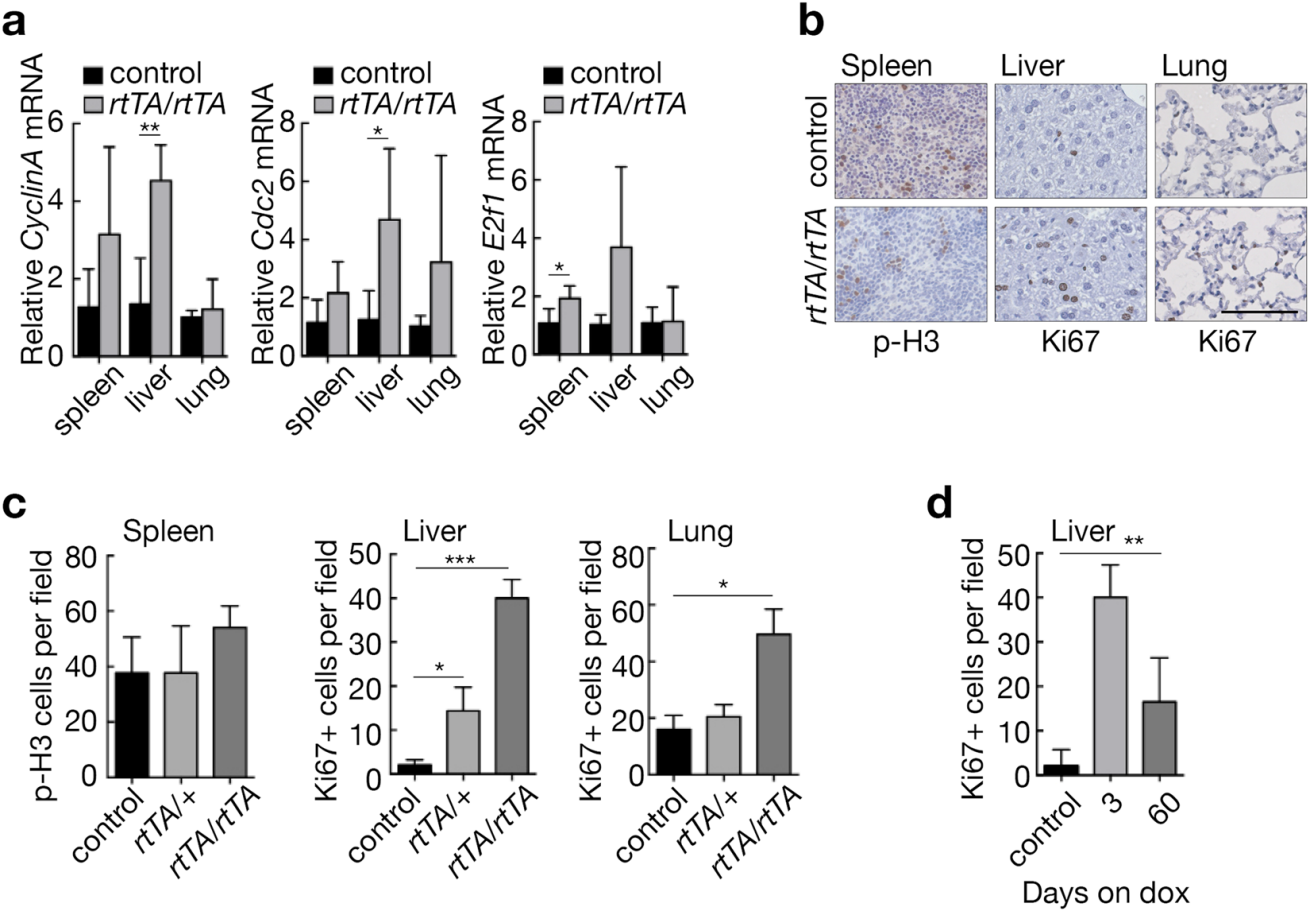
Consequences of acute *E2f3* induction in adult tissues. (**a**) Quantitative RT-PCR analysis of *E2f1*, *Cdc2* and *CyclinA* in adult control (*n* = 5 (spleen, lung), n = 4 (liver)) and *E2f3* ^*TRE/TRE*^; *Rosa26* ^*rtTA/rtTA*^ (*n* = 3) mouse tissues following administration of doxycycline for three days. Expression is normalized to *HPRT* and relative to the mean of the control samples within each tissue. Error bars, s.e.m. Two-tailed t-test; control vs *rtTA/*rtTA: **P = 0.01 (*CyclinA*, liver), 0.05* (*Cdc2*, liver), 0.05* (*E2f1*, spleen). (**b**) Immunohistochemical staining of phosphorylated histone 3 (p-H3) in the spleen and Ki67 in the liver and lung of control and *E2f3* ^*TRE/TRE*^; *Rosa26* ^*rtTA/rtTA*^ mice following administration of doxycycline for three days. Scale bar 100 µm. (**c**) Quantification of p-H3 positive nuclei per field of view of the spleen (red pulp) or Ki67 positive nuclei per field of view in the liver (hepatocytes) and lung isolated from control (*n* = 5(spleen, lung), n = 9(liver)), *E2f3* ^*TRE/TRE*^; *Rosa26* ^*rtTA/*+^ (*n* = 3(spleen), n = 4(liver, lung)) and *E2f3* ^*TRE/TRE*^; *Rosa26* ^*rtTA/rtTA*^ (*n* = 4(spleen, lung), n = 3(liver)) mice following administration of doxycycline for three days. Mean of 5 images per mouse; error bars, s.e.m. Mann-Whitney test; control vs. rtTA/+, *P = 0.03 (liver); control vs. rtTA/rtTA, ***P = 0.005 (liver), 0.03* (lung). (**d**) Quantification of Ki67 positive nuclei per field of view in liver (hepatocytes) isolated from control (*n* = 9) and *E2f3* ^*TRE/TRE*^; *Rosa26* ^*rtTA/rtTA*^ mice after 3 (*n* = 3) or 60 (*n* = 5) days continuous doxycycline administration. Mean of 5 images per mouse; error bars, s.e.m. Control and rtTA/rtTA quantifications from panel C are included for comparison. Mann-Whitney test; control vs. 60days, **P = 0.005. Controls for (a–d) include *E2f3*﻿^*TRE/TRE*^﻿ on and off doxycycline and *E2f3* ^*TRE/TRE*^; *Rosa26* ^*rtTA/+*^ and *E2f3* ^*TRE/TRE*^; *Rosa26* ^*rtTA/rtTA*^ maintained in the absence of doxycycline.

Consistent with its role in driving cell cycle, enforced expression of endogenous *E2f3* in *E2f3* ^*TRE/TRE*^; *Rosa26* ^*rtTA/rtTA*^ mice induced known E2F3 target genes, *Ccna1, Cdc2*, and *E2f1* (Fig. 4a) in all tissues examined (spleen, liver, and lung). Increased proliferation (Ki67- or p-H3-positive cells) was clearly evident within 3 days of enforced *E2f3* expression (Fig. 4b,c) in two relatively quiescent adult tissues, lung and liver while in spleen, which is already highly proliferative, a modest increase was also evident. The increase in proliferation, which was rtTA level-dependent (i.e. greater in 2 *rtTA* alleles versus 1) (Fig. 4c), is consistent with the increase in proliferation induced by classical transgenic over-expression of ectopic *E2f3* expression in other mouse models^16–18, 32^. Interestingly, E2F3-induced proliferation in liver initially peaked and then fell back to a lower, although still elevated, level that was thereafter sustained for at least 60 days, so long as *E2f3* induction was maintained (Fig. 4d). Similarly, peripheral leukocyte populations showed a trend towards a transient increase immediately following ectopic E2f3 expression (Supplementary Fig. 4). Hence, acute induction of enforced *E2f3* expression drives ectopic proliferation in multiple somatic tissues of adult mice.

**Figure 5:**
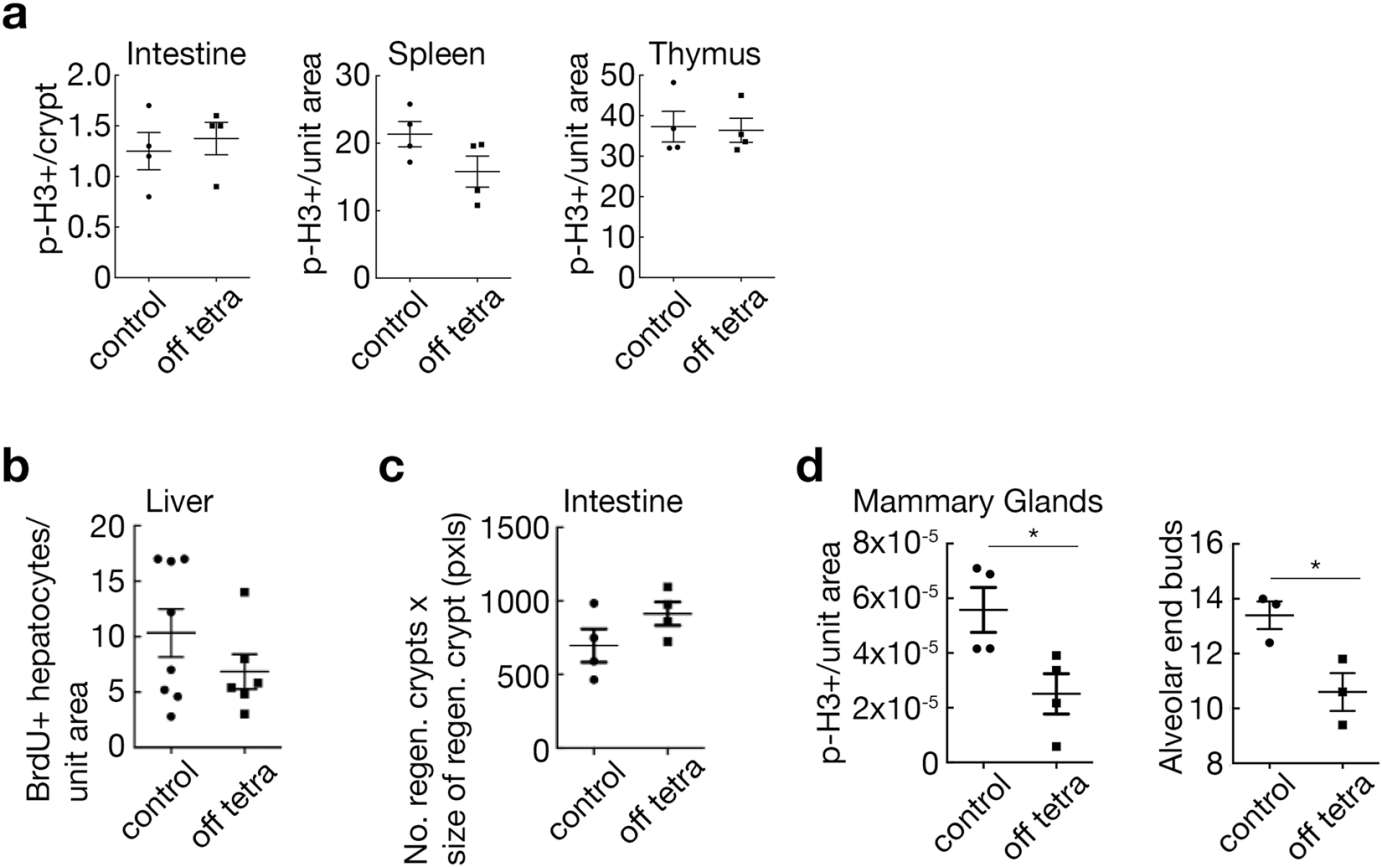
Consequences of *E2f3* repression in adult tissues. (**a**) Quantification of p-H3 positive nuclei per intestinal crypt or field of view of spleen and thymus in control *E2f3* ^*TRE/TRE*^; *βactin-tTS* mice maintained on 50 mg/L tetracycline and control *E2f3* ^*TRE/TRE*^ mice and *E2f3*-repressed *E2f3* ^*TRE/TRE*^; *β-actin-tTS* mice following 1 or 2 weeks withdrawal of tetracycline. *n* = 4 mice, mean of 5 images per mouse; error bars, s.e.m. Mann-Whitney test; *P* = 0.63(intestine), 0.23(spleen), 0.83(thymus). (**b**) Quantification of hepatocytes with BrdU positive nuclei per field of view in control (*n* = 8) and *E2f3* ^*TRE/TRE*^; *β-actin-tTS* (*n* = 6) mice maintained in the absence of tetracycline for a minimum of 3 weeks (off tetra) and 3 days post induction of liver damage with CCl_4_. Mean of 5 images per mouse; error bars, s.e.m. Mann-Whitney test; *P* = 0.48. (**c**) Intestinal regeneration was calculated by counting the number of viable crypts and multiplying that by the average size of the regenerating crypts^50^ in control *E2f3* ^*TRE/TRE*^ and *E2f3* ^*TRE/TRE*^; *β-actin-tTS* mice several weeks post withdrawal of tetracycline (off tetra) and 3 days post exposure to 14 Gy of ionizing radiation. *n = 4* mice, mean of 5 images per mouse; error bars, s.e.m. Mann-Whitney test; *P* = 0.34. (**d**) Quantification of p-H3 positive nuclei per unit area of epithelial cells in mid-gestation (E10.5) *E2f3* ^*TRE/TRE*^ and *E2f3* ^*TRE/TRE*^; *β-actin-tTS* mice maintained for several weeks in the absence of tetracycline. *n* = 3 mice, mean of 5 images per mouse; error bars, s.e.m. Mann-Whitney test; *P = 0.03. Quantification of the number of terminal end buds per 5x field collected from mid-gestation (E10.5) *E2f3* ^*TRE/TRE*^ and *E2f3* ^*TRE/TRE*^; *β-actin-tTS* mice maintained for several weeks in the absence of tetracycline. *n* = 3 mice, mean of 3–5 images per mouse. Two-tailed t-test; *P value = 0.03. Controls for (a–d) include *E2f3* ^*TRE/TRE*^ both on and off tetracycline and *E2f3* ^*TRE/TRE*^; *β-actin-tTS* mice maintained on 50 mg/L tetracycline.

By contrast, systemic repression of *E2f3* for one or two weeks in adult mice had no discernible impact on the proliferative indices of any tested tissues, including constitutively proliferating tissues such as small intestine, spleen and thymus (Fig. 5a). Likewise, expression of the common *E2f* target genes *Ccna1, Cdc2* or *E2f1* in multiple tissues was unperturbed by E2F3 repression (Supplementary Fig. 5). Furthermore, long-term systemic repression of *E2f3*, over many weeks, had no discernible impact on the health of animals, whose tissues remained histopathologically indistinguishable from those of wild-type controls (data not shown). These data are consistent with the notion that the function of E2F3 in adult tissue homeostasis is largely dispensable, presumably due to functional redundancy with other activator E2F family members^3, 5, 33^. To determine whether such functional redundancy remains sufficient to compensate for a lack of E2F3 activity under conditions of increased proliferative burden, we assessed the impact of E2F3 repression in acutely regenerating tissues. We saw no measurable inhibitory impact of E2F3 repression on liver regeneration after CCl_4_ injury or intestinal crypt regeneration after radiation injury (Fig. 5b,c). By contrast, mammary gland expansion and elaboration in pregnant mice was significantly retarded, as evidenced by decreased proliferation and reduced terminal budding (Fig. 5d). We conclude that, while E2F3 clearly contributes to the proliferative capacity of normal proliferating and regenerating tissues, its repression in most adult tissues can be complemented by alternate mechanisms – presumably E2F1 and E2F2 – even *in extremis* (see below).

### Determining real-time dependency on activator E2F activity in adult tissues *in vivo*

The common transcriptional activity of the three mitogenic E2Fs is essential for somatic cell cycle progression and proliferation of diverse cell types^7, 33–36^. However, the redundant functionalities of the mitogenic E2Fs - E2F1, E2F2, and E2F3 - obscure the impact of blocking any individual member (see above and ref. 4, 5), necessitating inhibition of all three to understand their shared role *in vivo*. Unfortunately, *E2f1-3* triple knock-out mice display embryonic lethality, as well as aberrant dynamics in multiple embryonic tissues – most notably, constitutively high levels of apoptosis^34, 36, 37^ – which precludes meaningful analysis of their shared role in adult tissue homeostasis and pathology. Therefore, to determine directly the role of global E2F1-3 activity in adult mice, we generated *E2f1* ^−/−^; *E2f2* ^−/−^; *E2f3* ^*TRE/TRE*^; *β-actin-tTS* mice in which, due to deficiency of E2F1 and E2F2, all activator E2F function is funnelled through the regulatable *E2f3* gene.

**Figure 6:**
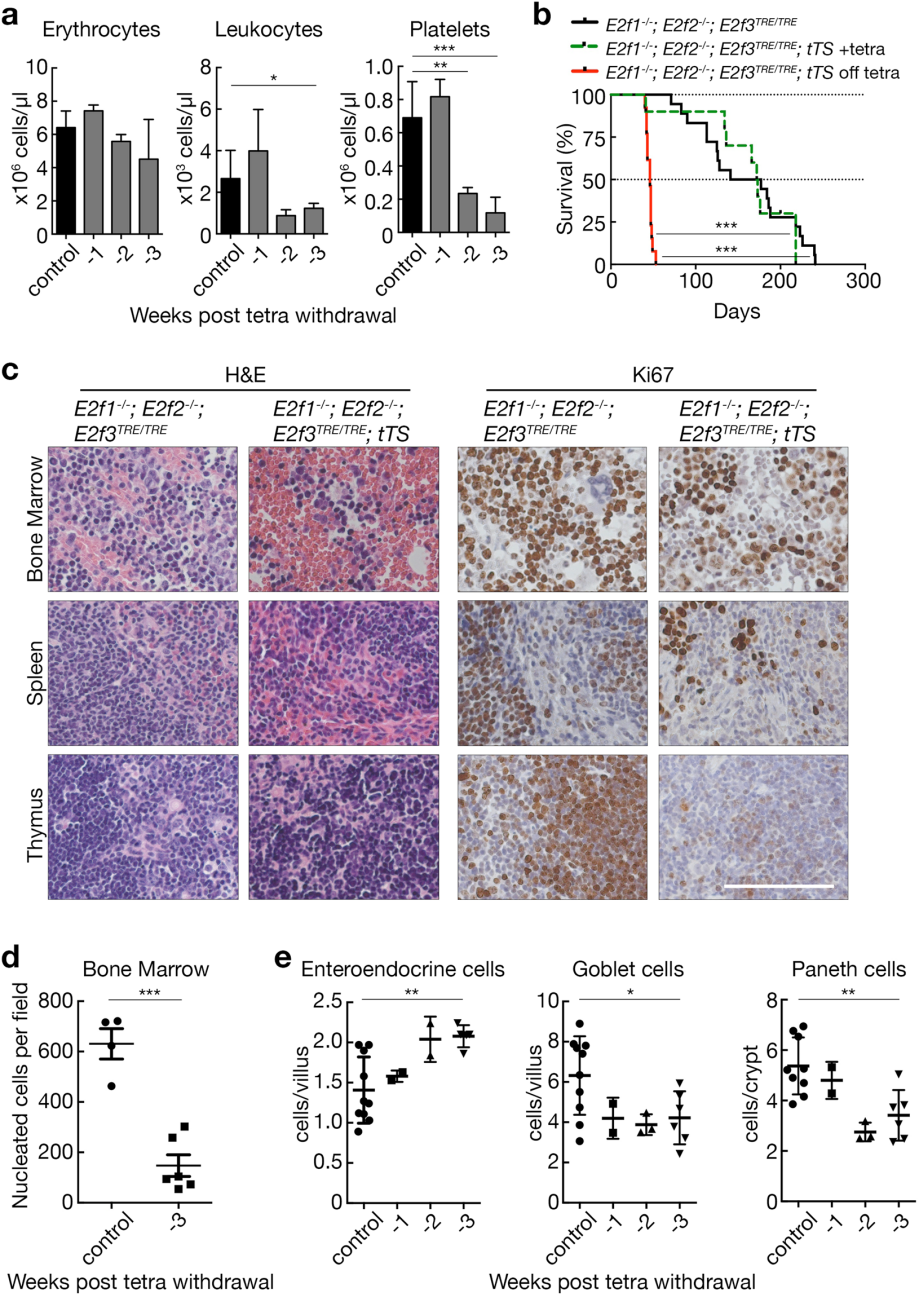
Repression of *E2f3* in the absence of *E2f1* and *E2f2* is rapidly lethal in adult mice. (**a**) Histogram of erythrocytes, leukocytes, and platelets in *E2f1* ^−/−^; *E2f2* ^−/−^; *E2f3* ^*TRE/TRE*^ control (*n* = 8) and *E2f1* ^−/−^; *E2f2* ^−/−^; *E2f3* ^*TRE/TRE*^; *β-actin-tTS* mice following withdrawal of 100 mg/L tetracycline for 1 (−1, *n* = 3), 2 (−2, *n* = 3(erythrocytes), n = 2(leukocytes, platelets)) or 3 (−3, *n* = 5(erythrocytes, platelets), n = 4(leukocytes)) weeks. Error bars, s.e.m. Student’s two-tailed t-test; control vs. -2weeks, **P = 0.007 (platelets); control vs. -3weeks, *P = 0.027 (leukocytes); control vs. -3weeks, ***P < 0.0001 (platelets). (**b**) Survival curve of *E2f1* ^−/−^; *E2f2* ^−/−^; *E2f3* ^*TRE/TRE*^ control (*n* = 18) and *E2f1* ^−/−^; *E2f2* ^−/−^; *E2f3* ^*TRE/TRE*^; *β-actin-tTS* mice maintained in the presence of tetracycline (+tetra, *n* = 10) or following removal of tetracycline (off tetra, *n* = 13). Log-rank (Mantel-Cox) test; control vs. off tetra, ***P < 0.0001; +tetra vs. off tetra, ***P = 0.0006. (**c**) Haematoxylin and eosin (H&E) stained images and Ki67 expression in bone marrow, spleen, and thymus in *E2f1* ^−/−^; *E2f2* ^−/−^; *E2f3* ^*TRE/TRE*^ control and *E2f1* ^−/−^; *E2f2* ^−/−^; *E2f3* ^*TRE/TRE*^; *β-actin-tTS* mice 3 weeks after withdrawal of tetracycline. Scale bar 100 µm. Representative images from *n*≥4 mice. (**d**) Quantification of nucleated cells per 40x field of view of the bone marrow of *E2f1* ^−/−^; *E2f2* ^−/−^; *E2f3* ^*TRE/TRE*^ control (*n* = 4) and *E2f1* ^−/−^; *E2f2* ^−/−^; *E2f3* ^*TRE/TRE*^; *β-actin-tTS* (*n* = 6) mice 3 weeks after withdrawal of tetracycline. (**e**) Quantification of enteroendocrine (Chromogranin A positive), goblet (Alcian blue positive) and Paneth (lysozyme positive) cells per intact villus or per intact crypt. In all cases, a total of 30 crypts/villi were counted per mouse from the small intestine of *E2f1* ^−/−^; *E2f2* ^−/−^; *E2f3* ^*TRE/TRE*^ control (n = 10(enteroendocrine, goblet), n = 9(Paneth)) and *E2f1* ^−/−^; *E2f2* ^−/−^; *E2f3* ^*TRE/TRE*^; *β-actin-tTS* mice following the indicated number of weeks (-1 weeks, n = 2), (-2 weeks, n = 2(enteroendocrine), n = 3 (goblet, Paneth)), (-3 weeks, n = 5(enteroendocrine), n = 6 (goblet, Paneth)) post withdrawal of tetracycline. Mann-Whitney test, control vs. -3 weeks, **P = 0.004 (enteroendocrine), 0.04* (goblet), 0.005** (Paneth).

*E2f1* ^−/−^; *E2f2* ^−/−^; *E2f3* ^*TRE/TRE*^; *β-actin-tTS* mice maintained on tetracycline (tet repressor inactive) were grossly and histopathologically indistinguishable from control *E2f1* ^−/−^; *E2f2* ^−/−^; *E2f3* ^*TRE/TRE*^ animals (data not shown) and exhibited similar life-spans (median survival: 172.5 and 159 days, respectively). However, withdrawal of tetracycline (tet repressor active) rapidly triggered profound pathologies. By three weeks post tetracycline withdrawal, *E2f1* ^−/−^; *E2f2* ^−/−^; *E2f3* ^*TRE/TRE*^; *β-actin-tTS* mice exhibited severe leukopenia and thrombocytopenia (Fig. 6a). Correlating with these reduced hematopoietic cell numbers in the periphery, reduced proliferation was evident in the bone marrow, spleen, and thymus (Fig. 6c) and reduced cellularity was observed in the bone marrow (Fig. 6d). The three main hematopoietic populations of the bone marrow – B-cells, T-cells, and myeloid cells – were all markedly reduced by three weeks post tetracycline withdrawal (Supplementary Fig. 6). At this time, the internal structures and relative geographical locations of differentiated cell populations within the spleen remained similar in both *E2f1* ^−/−^; *E2f2* ^−/−^; *E2f3* ^*TRE/TRE*^; *β-actin-tTS* mice and appropriate controls (Supplementary Fig. 7), but both the spleen and thymus appear macroscopically smaller in *E2f1* ^−/−^; *E2f2* ^−/−^; *E2f3* ^*TRE/TRE*^; *β-actin-tTS* mice than their controls (data not shown). By five weeks’ post tetracycline withdrawal, all mice were moribund and severely cytopenic, including erythrocytopenia (Fig. 6b and Supplementary Fig. 8). In the small intestine, acute repression of *E2f3* in *E2f1/2* doubly-deficient mice triggered a progressive increase in enteroendocrine cells and decrease in goblet and Paneth cells (Fig. 6e and Supplementary Fig. 9). Repression of *E2f3* in *E2f1* ^−/−^; *E2f2* ^−/−^ doubly deficient mice had no discernible impact on non-proliferating tissues such as liver, lung and kidney (data not shown). Hence, acute and long-term homeostatic dependency on functional E2F1-3 is restricted to proliferative organs and most marked in hematopoietic tissues.

**Figure 7:**
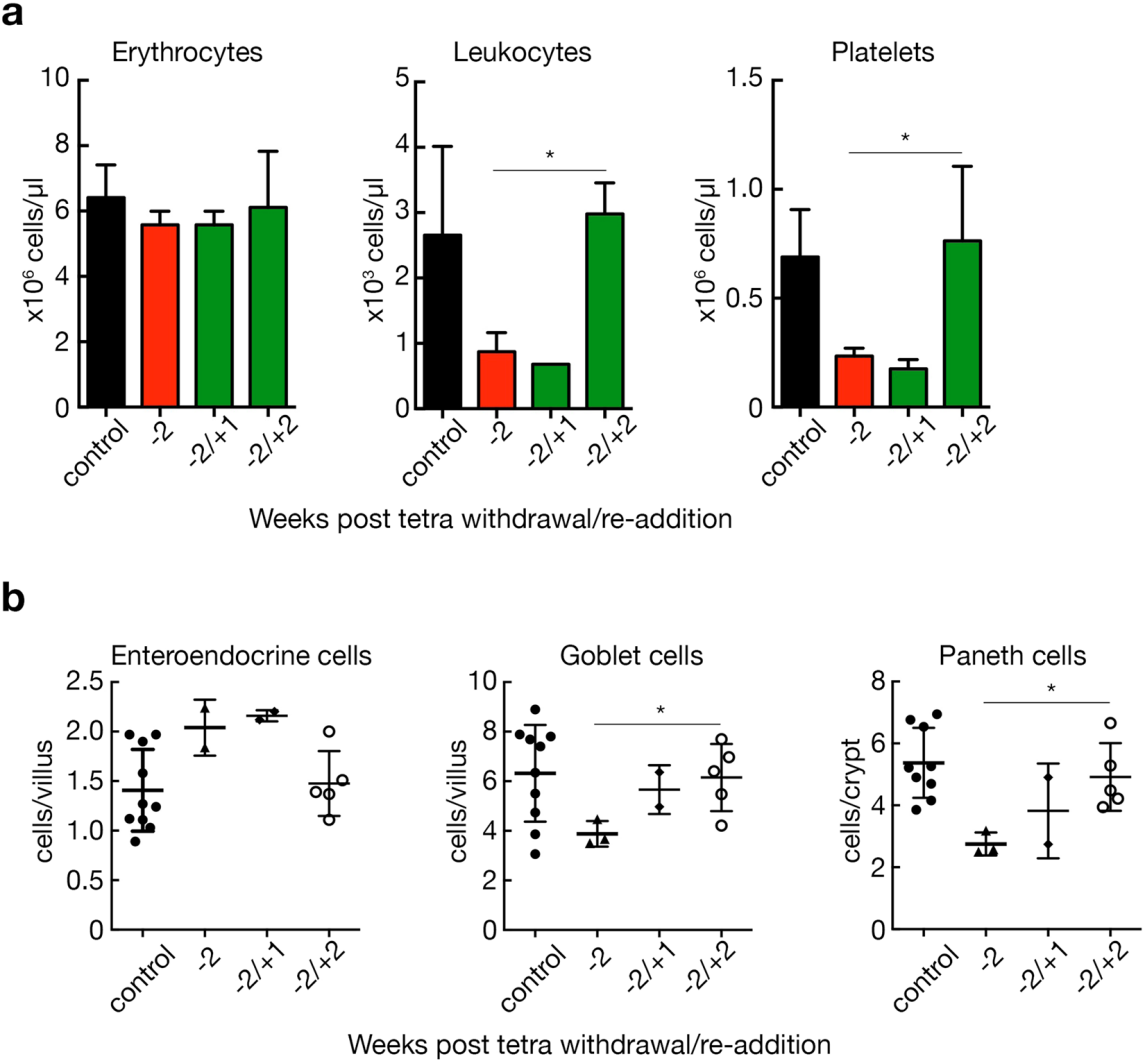
The deleterious effects mediated via the repression of activator E2Fs can be reversed within a limited time frame. (**a**) Histogram of total numbers of erythrocytes, leukocytes, and platelets in in *E2f1* ^−/−^; *E2f2* ^−/−^; *E2f3* ^*TRE/TRE*^ control (n = 8) and *E2f1* ^−/−^; *E2f2* ^−/−^; *E2f3* ^*TRE/TRE*^; *β-actin-tTS* mice following withdrawal of 100 mg/L tetracycline for 2 weeks (−2, n = 3), or following removal of tetracycline for 2 weeks followed by re-administration of tetracycline for the indicated number of weeks (−2/+1, n = 3(erythrocytes), n = 2(leukocytes, platelets)) and (−2/+2, n = 5(erythrocytes, platelets), n = 4(leukocytes)), respectively. Error bars, s.e.m. Two-tailed t-test; -2weeks vs. −2/+2weeks, **P = 0.001(leukocytes), 0.04*(platelets). (**b**) Quantification of differentiated intestinal cells per villus or crypt (stained as in Fig. 5D) in *E2f1* ^−/−^; *E2f2* ^−/−^; *E2f3* ^*TRE/TRE*^ control (n = 10(enteroendocrine, goblet), n = 9(Paneth)) and *E2f1* ^−/−^; *E2f2* ^−/−^; *E2f3* ^*TRE/TRE*^; *β-actin-tTS* mice following tetracycline removal for 2 weeks (-2, n = 2(enteroendocrine), n = 3 (goblet, Paneth)), or following removal of tetracycline for 2 weeks followed by re-administration of tetracycline for the indicated number of weeks (−2/+1, n = 2), (−2/+2, n = 5). Two-tailed t-test; -2weeks vs. −2/+2weeks, *P = 0.04 (goblet), 0.02*(Paneth).

Given the dramatic and rapidly lethal impact of *E2f1-3* repression in *E2f1* ^−/−^; *E2f2* ^−/−^; *E2f3* ^*TRE/TRE*^; *β-actin-tTS* mice, we next asked whether expeditious reversal of *E2f3* repression could reverse the blood and intestinal phenotypes and preserve viability. Indeed, re-administration of tetracycline to leukopenic *E2f1* ^−/−^; *E2f2* ^−/−^; *E2f3* ^*TRE/TRE*^; *β-actin-tTS* mice deprived of tetracycline for two weeks rapidly and completely reversed both cytopenic and intestinal phenotypes, leading to full recovery (Fig. 7a,b and Supplementary Fig. 9). By contrast, re-administration of tetracycline to near moribund *E2f1* ^−/−^; *E2f2* ^−/−^; *E2f3* ^*TRE/TRE*^; *β-actin-tTS* mice deprived of tetracycline for three weeks failed to rescue the animals (data not shown). These data define a tight temporal window during which E2F1-3-deprived mice can recover if E2F function is restored.

## Discussion

We describe a novel class of switchable genetic mouse model, using the *E2f3* gene as an exemplar, which allows for reversible up or down regulation of an endogenous gene in adult mice at will. Such regulation is achieved by inserting a TRE into the endogenous *E2f3* promoter, allowing external modulation of the endogenous *E2f3* gene via either a tetracycline-dependent transcriptional activator (rtTA) or a tetracycline-dependent repressor tTS(kid) or rtTS. Insertion of the TRE by itself has no significant effect on the normal regulation of *E2f3* aside from a very mild decrease of *E2f3* expression in the livers of *E2f3* ^*TRE/TRE*^ mice. Nonetheless, there is an obvious requirement to avoid disrupting as many cis-regulatory sequences as possible when choosing the site for TRE insertion, as illustrated by the instance of TRE insertion into the *Hoxa2* 5′ untranslated region, which resulted in markedly decreased expression of *Hoxa2* ^38^. In our *E2f3* case, TRE insertion into the promoter proved highly effective for ectopic *E2f3* regulation. However, as indicated by other studies involving ectopic regulation of endogenous genes^39^, each targeted gene will need to be considered individually, most especially when seeking rtTA-dependent transcriptional activation.

Expression of the rtTA transcriptional activator in *E2f3* ^*TRE*^ mice resulted in dramatically elevated and persistent doxycycline dependent ectopic *E2f3* expression, which was reversible upon withdrawal of doxycycline. Encouragingly, analysis of *E2f3’s* two neighbouring genes, *Mboat1* and *Cdkal1*, showed them to be unaffected by either TRE insertion itself or by the rtTA-dependent upregulation of *E2f3*, indicating the high precision of *E2f3* gene activation. Of note, the extent of *E2f3* induction was around two-fold higher in animals carrying two versus one rtTA alleles, further embellishing the tunability of the experimental system. Such doxycycline-dependent upregulation resembles that seen in a previous study involving upregulation of a TRE-modified *Mlc1* allele and an alternative tTA system^40^.

Conversely, expression of the tTS(kid) or rtTS repressors in *E2f3* ^*TRE*^ cells and tissues allows for profound, doxycycline-dependent, reversible repression of endogenous *E2f3*. Hence, the combined iterations of these mouse models allow for the reversible toggling of endogenous *E2f3*, up and down, at will, in adult tissues. Of note, in both of our *E2f3* activation and repression models, *E2f3a* and *E2f3b* isoforms are both co-regulated. Hence, in this study we cannot attribute any phenotype to a particular E2F3 isoform but rather to total E2F3. However, while we acknowledge that E2F3a and E2F3b possess some distinct properties, past studies indicate that each can significantly complement for the absence of the other^14^.

We generated two iterations of the TRE-dependent *E2f3* repression system, one (*E2f3* ^*TRE/TRE*^; *β-actin-tTS*) repressing *E2f3* upon tetracycline withdrawal and the other (*E2f3* ^*TRE/TRE*^; *Rosa26* ^*CAG-rtTS/*+^) repressing *E2f3* upon doxycycline addition. Each iteration has its own advantages in terms of rapidity of switching, building up of antibiotic reservoirs in bone and teeth over extended antibiotic administration, and minimizing perturbation of animals. Either addition of doxycycline to *E2f3* ^*TRE/TRE*^; *Rosa26* ^*CAG-rtTS/*+^ mice or withdrawal of tetracycline from *E2f3* ^*TRE/TRE*^; *tTS* adult mice triggered rapid repression of endogenous *E2f3* expression in all tested tissues. *E2f3* ^*TRE/TRE*^; *β-actin-tTS* embryos born to mothers maintained on doxycycline were born with Mendelian frequency. However, the embryos of the same genotype developed in mothers deprived of doxycycline failed to survive to birth and exhibited the same heart trabeculation deficit as that seen in classical *E2f3* knockout embryos. Essentially complete *E2f3* repression in doxycycline-deprived *E2f3* ^*TRE/TRE*^; *β-actin-tTS* embryos was confirmed by analysis of E2F3 protein expression isolated from *E2f3* ^*TRE/TRE*^; *β-actin-tTS* MEFs; E2F3 protein is normally expressed in doxycycline-treated *E2f3* ^*TRE/TRE*^; *β-actin-tTS* MEFs but completely absent from doxycycline-deprived fibroblasts. Intriguingly, subsequent *in vitro* addition of doxycycline to MEFs in which *E2f3* had been repressed throughout development failed to restore E2F3a/b expression (Supplementary Fig. 10), indicating that the *E2f3* gene is permanently silenced if actively repressed through embryogenesis. The mechanism behind this is unclear but may involve methylation, as observed elsewhere^41^.

In adult tissues of *E2f3* ^*TRE/TRE*^; *β-actin-tTS* mice, *E2f3* repression was effectively complete and maximal by 1 week of antibiotic withdrawal, persisting thereafter for as long as tetracycline was withheld. Despite the profound repression of *E2f3* expression in tetracycline-deprived *E2f3* ^*TRE/TRE*^; *β-actin-tTS* mice, we nonetheless observed little impact on proliferation rates of tissues, their architecture and histology, or expression of key E2F1-3 transcriptional targets, such as *E2f1*, *Cdc2* and *Cyclin A*. This lack of overt phenotype was true even after extended (9 week) absence of tetracycline. In contrast to our inability to reverse constitutive repression through embryonic development, *E2f3* repression acutely imposed in adult tissues proved rapidly reversible upon re-administration of tetracycline.

While the lack of any phenotype associated with acute E2F3 repression is unsurprising in non-proliferating tissues like lung and liver, we also saw no measurable impact in proliferative tissues like intestine, spleen and thymus. Equally surprisingly, *E2f3* repression had no inhibitory impact on proliferation in tissues regenerating after damage – specifically liver after CCl_4_ injury and intestine after irradiation. Only in the mammary tissue of pregnant mice was any impact of acute *E2f3* repression evident, which manifested as the significant retardation of terminal end bud proliferation and elaboration. This observation may be relevant given the previously published potentiating role of *E2f3* in experimental mammary cancers of mice^11, 12^.

Previous reports of tTS-mediated repression of endogenous genes *in vivo* are largely limited to developmental processes^42, 43^ and utilized doxycycline to inactivate the tTS repressor. One technical issue we encountered with the *E2f3* ^*TRE/TRE*^; *β-actin-tTS* system was the tardy onset of repression because of the relatively long biological half-life of doxycycline, in part a consequence of developmental accumulation of the antibiotic in somatic reservoirs, most notably bone and teeth. To minimize these problems, we used tetracycline in place of doxycycline in all of our tTS repression studies: tetracycline has shorter plasma half-life and proved effective at doses as low as 50 mg/L. To circumvent accumulation of the antibiotic, we also reconfigured the tTS(kid) repressor, which is inactivated by tetracycline, to the corresponding reverse tetracycline rtTS(kid) variant, which is dependent on the drug for its activity. This *Rosa26* ^*CAG-rtTS*^ strain is ideal for studies in which normal E2F3 expression is needed during development, followed by acute E2F3 repression in adult tissues. Furthermore, the new *Rosa26* ^*CAG-rtTS*^ allele exists in a conditional form in which rtTS protein expression is dependent upon Cre activity, allowing repression to be manifest only within specific target cell-types or tissues. Similar conditional rtTA alleles are readily available for complementary overexpression studies. These further refinements to the experimental system significantly enhance its utility and flexibility.

The most plausible explanation for the mildness of the *E2f3*-repression phenotype is complementation by the two other activator E2Fs, E2F1 and E2F2^3, 5, 7, 33^. To investigate this redundancy, we crossed our *E2f3* ^*TRE/TRE*^; *β-actin-tTS* mouse into an *E2f1* ^−/−^; *E2f2* ^−/−^ double knockout background, effectively constraining all activator E2F activity to channel through the switchable *E2f3* ^*TRE*^ allele. In this way, we could evaluate the role of E2F1 and E2F2 in compensating for loss of E2F3 function and also gauge the systemic impact of transiently shutting down all activator E2F activity on tissues. Systemic blockade of activator E2F1-3 activity triggered dramatic changes in differentiation patterns within the small intestine, precipitating a progressive accumulation of enteroendocrine cells and loss of goblet and Paneth cells, despite having no discernible impact on proliferation. A previous report using a β-napthoflavone-driven conditional knock-out strategy similarly suggested that E2F1-3 activities are dispensable for adult intestinal proliferation and homeostasis^34^. However, conditional knockout targeting is irreversible, critically dependent upon both the promoter used to drive Cre expression and variable target cell recombination efficiencies, and necessarily involves CRE-recombinase–induced DNA damage response^44–47^, all of which are circumvented by the global, reversible *E2f3* repression achievable with the *E2f3* ^*TRE/TRE*^; *β-actin-tTS* or *E2f3* ^*TRE/TRE*^; *Rosa26* ^*CAG-rtTS*^ system.

Repressing *E2f3* in *E2f1* ^−/−^; *E2f2* ^−/−^ doubly-deficient mice triggered profound collapse of bone marrow, together with the onset of leukopenia, thrombocytopenia and progressive erythrocytopenia, with corresponding decreases in lymphocyte and myeloid cell populations. These data are consistent with attrition of each blood cell type according to its natural lifespan. Mice were moribund following 3 weeks post tetracycline withdrawal. However, re-administration of tetracycline two weeks after its withdrawal, at a time of rapidly declining blood counts, reversed all pathologies and all animals completely recovered. Such complete rescue of all animals by timely restoration of activator E2F activity defines a potentially useful therapeutic window for any treatment modality that works by blocking activator E2F activity.

## Materials and Methods

### Generation of *E2f3*^*TRE*^ mice

A vector containing 15 kb of *E2f3* genomic sequence was a kind gift from Jacqueline Lees. The TRE element was isolated from pTre2 (Clontech) using KpnI and XhoI, and then cloned into a KpnI site 500 bp upstream of the *E2f3*a translation start site along with a LoxP flanked Neomycin resistance cassette. No *E2f3* sequences were removed or altered at the insertion site, although silent mutations were incorporated into the first exons of *E2f3a* and *E2f3b*. The final sequences encompassing these mutations is as follows: *E2f3aWT*: cggtggcccaccg/*E2f3aMut*: TggAggAccTccg; *E2f3bWT*: cggaaatgcccttacagcagcag/*E2f3b*Mut: cggaaatgccACtTcaAcagcag. Finally, a DTA cassette was added to this vector to allow negative selection in mouse embryonic stem cells. This vector was then linearized using NotI and introduced into *Sv/129* mouse embryonic stem cells. Correct targeting was confirmed by Southern blot, and correctly targeted clones were transiently transfected with a plasmid expressing Cre-recombinase in order to remove the conditional Neomycin resistance cassette. The final sequence inserted to the KpnI site of mESCs used to generate chimeric animals is below; the residual single LoxP site is in bold:

GGTACCCGGGGATCCTCTAGACTCGAGGAATTCCGATCATATTCAATAACCCTTAAT**ATAACTTCGTATAATGTATGCTATACGAAGTTAT**TAGGTCTGAAGAGGAGTTTACGTCCAGCCAAGCTAGCTVTGGCTGCAGGTCGAGTTTACCACTCCCTATCAGTGATAGAGAAAAGTGAAAGTCG AGTTTACCACTCCCTATCAGTGATAGAGAAAAGTGAAAGTCGAGTTTACCACTCCCTATCAG TGATAGAGAAAAGTGAAAGTCGAGTTTACCACTCCCTATCAGTGATAGAGAAAAGTGAAAGT CGAGTTTACCACTCCCTATCAGTGATAGAGAAAAGTGAAAGTCGAGTTVTACCACTCCCTATC AGTGATAGAGAAAAGTGAAAGTCGAGTTTACCACTCCCTATCAGTGATAGAGAAAAGTGAAAGTCGAGCTCGGTACC.

*Sv/129* mESCs were injected into C57/B6 oocytes and chimeras were evaluated by coat colour. Germline transmission was confirmed by PCR and verified by Southern analysis.

### Generation of *Rosa26*^*CAG-rtTS*^ mice

Overlap extension PCR was used to clone the tetracycline responsive domain from rtTA*M2 (Clontech, pTet-on Advanced) to the KRAB-AB silencing domain of the Kid-1 protein (SDKid-1) (Clontech, pTet-tTS), creating the chimeric rtTS protein. The full sequence encoding the chimeric protein is:

ATGTCTAGACTGGACAAGAGCAAAGTCATAAACGGCGCT CTGGAATTACTCAATGGAGTCGGTATCGAAGGCCTGACGACAAGGAAACTCGCTCAAAAGCTGGGAGTTGAGCAGCCTACCCTGTACTGGCACGTGAAGAACAAGCGGGCCCTGCTCGATGCCCTGCCAATCGAGATGCTGGACAGGCATCATACCCACTTCTGCCCCCTGGAAG GCGAGTCATGGCAAGACTTTCTGCGGAACAACGCCAAGTCATTCCGCTGTGCTCTCCTCTCACATCGCGACGGGGCTAAAGTGCATCTCGGCACCCGCCCAACAGAGAAACAGTACGAAACCCTGGAAAATCAGCTCGCGTTCCTGTGTCAGCAAGGCTTCTCCCTGGAGAACGCACTGTACGCTCTGTCCGCCGTGGGCCACTTTACACTGGGCTGCGTATTGGAGGAACAGGAGCATCAAGTAGCAAAAGAGGAAAGAGAGACACCTACCACCGATTCTATGCCCCCACTTCTGAGACA AGCAATTGAGCTGTTCGACCGGCAGGGAGCCGAACCTGCCTTCCTTTTCGGCCTGGAACTAATCATATGTGGCCTGGAGAAACAGCTAAAGTGCGAAAGCGGCGGGCCAAAAAAGAAGAGAAAGCTAGCAGTGTCAGTGACATTTGAAGATGTGGCTGTGCTCTTTACTCGGGACGAGTGGAAGAAGCTGGATCTGTCTCAGAGAAGCCTGTACCGTGAGGTGATGCTGGAGAATTACAGCAACCTGGCCTCCATGGCAGGATTCCTGTTTACCAAACCAAAGGTGATCTCCCTGTTGCAGCAAGGAGAGGATCCCTGGTAAA.

The SA70b pROSA-CAGGS-attP50/B53-hygro-NLSLacZ vector was obtained from ARTEMIS Pharma-ceuticals. A fragment containing the CAGGS promoter and an intron was removed from SA70b and cloned into a modified pROSA26 plasmid^48^, creating the pROSA26CAG-PAS vector. The rtTS cDNA above was cloned into pBigT, and then into pROSA26CAG-PAS^48^ to create the final *Rosa26* ^*CAG-LSL-rtTS*^ targeting vector. This vector was then linearized introduced into mouse embryonic stem cells. Germ-line transmission was confirmed by PCR and verified by Southern analysis. A *Rosa26* ^*CAG-LSL-rtTS/*+^ male was bred to a Pgk-Cre^49^ female which removed the Lox-stop-lox cassette in the germline to produce *Rosa26* ^*CAG-rtTS/*+^ mice, which were then intercrossed with *E2f3* ^*TRE/TRE*^.

### Mouse maintenance

All animals were kept under SPF conditions, and all experimental procedures approved by the University of California, San Francisco Institutional Animal Care and Use Committee or Home Office UK guidelines under project licenses to G.I.E. (70/7586, 80/2396) at the University of Cambridge and all experimental procedures were conducted in accordance with these guidelines and regulations. Where specified, animals were supplied with drinking water containing either tetracycline or doxycycline that was replenished twice per week in light-protected bottles. For tTS experiments, tetracycline hydrochloride (Sigma T7660) was dissolved at either 50 mg/L (for experiments with *E2f3* ^*TRE/TRE*^; *β-actin-tTS* mice) or 100 mg/L (for experiments with *E2f1* ^−/−^; *E2f2* ^−/−^; *E2f3* ^*TRE/TRE*^; *β-actin-tTS* mice)and doxycycline hyclate (Sigma D9891) was dissolved at 100 mg/L. For rtTA and rtTS experiments doxycycline hyclate was dissolved at 2 g/L. In all cases, doxycycline and tetracycline were dissolved in water containing 3% sucrose to increase palatability.

### Mouse genotyping

Ear biopsies were collected from 2–5 week old mice and digested overnight at 55 °C in 10% Chelex 100 Resin (Bio-Rad Catalog #142–1253), 0.1% Tween-20, and 0.25 mg/ml Proteinase K (Sigma P8044). Alternatively, tail biopsies were collected (UCSF) and digested overnight at 55 °C in tail lysis buffer (50 mM Tris, 50 mM EDTA, 0.5% SDS). gDNA was then precipitated using two volumes of 100% ethanol, washed once in 70% ethanol, and resuspended in water.

Genotyping primers for the *E2f3* ^*TRE*^ allele were designed to span the 300 bp insertion of the TRE element. Forward: 5′-CCAAAACCGAAACTTGCGCTCAAGAC-3′ and Reverse: 5′- GATACGGTTTACGCGCCAAGGTCCTC-3′. The wild-type allele gives a 424 bp band while a TRE targeted allele generates a 865 bp band. Genotyping primers for the *β-actin-tTS* allele are as follows: Forward: 5′- CCCAGAAGCTAGGTGTAGAGCA-3′ Reverse: 5′- GGCGGCATACTATCAGTAGTAGG-3′. General primers detecting *Rosa26CAG* were used to detect *Rosa26* ^*CAG-rtTS*^, with primer sequences as follows: Universal forward: 5′-CTCTGCTGCCTCCTGGCTTCT-3′ Wild-type reverse: 5′-CGAGGCGGATCACAAGCAATA CAG reverse: 5′ TCAATGGGCGGGGGTCGTT. A typical PCR reaction was performed using GoTaq (Promega) with 1 μl of Chelex extracted gDNA following manufacturers’ instructions. The following PCR conditions were applied: 5 min, 95 °C initial denaturation; 35 cycles of 30 s at 95 °C 30 at, 60 °C and 1.5 min at 72 °C, followed by a final 5 min at 72 °C. PCR amplification products were analyzed by agarose gel electrophoresis. Jackson Labs (https://www.jax.org/), provides genotyping information for *Rosa26rtTA* (*Gt*(*ROSA*)*26Sor* ^*tm1*(rtTA*M2)*Jae*^), *E2f1* knockout (*E2f1* ^*tm1Meg/J*^), and *E2f2* knockout (*E2f2* ^*tm1Zubi*^).

### Mouse Embryonic Fibroblasts

*E2f3* ^*TRE/TRE*^ and control wild-type mouse embryonic fibroblasts (MEFs) were generated from embryos 13.5 days after fertilization and cultured in DMEM (Thermo Fisher 41966052) supplemented with penicillin-streptomycin (Thermo Fisher, 15140-122), L-glutamine (Thermo Fisher, 25030-024), and BGS (Hyclone SH30541.03HI). All experiments were performed between passage 3 and 5. To render cells quiescent, MEFs were plated at 8 × 10^5^ cells per 6 cm culture dish in 10% BGS. The next day, MEFs were washed twice with PBS and then cultured in media containing 0.1% BGS for 72 hours. To stimulate cell-cycle re-entry, quiescent MEFs were stimulated with DMEM containing 20% BGS. For asynchronously cycling experiments, MEFs were plated at 3 × 10^5^ cells per 6 cm culture dish in 10% BGS and collected 48 hours later.

### qPCR

Total RNA was isolated using TRIzol Reagent (Thermo Fisher, 15596-018) following manufacturers instructions. Up to 1 μg of cDNA was synthesized using the High-Capacity cDNA Reverse Transcription Kit with RNase Inhibitor (Thermo Fisher, 4374966) following manufacturers instructions. qPCR reactions were performed on a Roche Lightcycler 480 using Lightcycler 480 DNA SYBR Green I mix or Taqman Universal Master Mix II, or an Applied Biosystems StepOne using Fast SYBR Green Master Mix (Thermo Fisher, 4385612), following manufacturers instructions. Primers used in combination with SYBR Green were *E2f3: forward-tacggagtcccgatagtcca, reverse-gaccccatcaggagactgg*; *E2f3a: forward-caaggaccctccagcagag, reverse-agttccagccttcgctttg*; *Ef3b: forward-gctttcggaaatgcccttac, reverse-ggtactgatggccactctcg*; *E2f1: forward-tgccaagaagtccaagaatca, reverse-cttcaagccgcttaccaatc*; *Cdc2: forward-agaaggtacttacggtgtggt, reverse-gagagatttcccgaattgcagt*; *CyclinA2: forward-gccttcaccattcatgtggat, reverse-ttgctgcgggtaaagagacag*; *HPRT: forward-ctggtgaaaaggacctctcgaag, reverse-ccagtttcactaatgacacaaacg*. For Taqman gene expression assays, the following commercial primer/probe sets were used: *Actin* (4352933E, Applied Biosystems), *Mboat1* (4331182, Applied Biosystems) and *Cdkal1* (4351372, Applied Biosystems).

### Immunoblotting

Animal tissues were ground into powder on liquid nitrogen and proteins extracted in buffer containing 1% SDS, 50 mM Tris pH6.8 and 10% Glycerol on ice for 10 minutes. Lysates were boiled for 10 minutes, followed by sonication (Bioruptor, Diagenode) for 15 minutes on high, with 30 second on/off cycles. Total protein (50 µg) was electrophoresed on an SDS-PAGE gel and blotted onto immobilon-P (Millipore) membrane. Membranes were blocked in 5% milk and primary antibodies incubated overnight at 4 °C. Secondary antibodies were applied for 1 hour followed by chemiluminescent visualization. Primary antibodies; E2F3 (Santa-Cruz Biotechnology, sc-878, used at 1:1000), GAPDH (Cell Signaling Technology, 5174 S, used at 1:5000), β-Actin (Santa-Cruz Biotechnology, sc-69879, used at 1:5000).

### Statistical analysis

Statistical analyses were performed using GraphPad Prism v6.0d (GraphPad Software, Inc., San Diego, CA, USA) as indicated with *P* ≤ 0.05 considered to be statistically significant.

### Quantification of mammary gland terminal end buds

To quantify mammary gland terminal end buds (TEBs), hematoxylin and eosin stained sections from paraffin embedded number four glands were analyzed. Low magnification pictures in proximity to the lymph node were taken. Transversely cut TEBs can be identified by the presence of multiple cell layers and a lack of a ductal lumen. These structures were counted in at least three independent biological repeats per genotype by two independent researchers.

### Blood cell analysis

Peripheral blood was collected into EDTA or heparin-coated tubes and 40 µl was analyzed on a Sysmex haematology analyser. For estimation of total cellularity of bone marrow, the total number of nucleated cells was counted per field of view from a hematoxylin and eosin stained section. For the B220, CD3, and Cd11b quantifications of cells within the bone marrow, the number of positively stained cells were counted per field of view. For both cellularity and cell surface marker stainings in the bone marrow, 3 images per organ/mouse were taken at 40x magnification and quantified; the mean of 3 raw counts was calculated and represents one data point per graph.

### Image acquisition tools and image processing software packages

Immunoblots were developed on Fuji RX X-ray film 18 × 24 cm and then scanned on an Epson Perfection V500 Photo flatbed scanner. Images were cropped using Adobe Photoshop, but were otherwise unprocessed. Immunohistochemical staining was imaged on a Zeiss Axio Imager using the Zeiss AxioVision 4.8 software using the AutoLive setting and interactive white balance. Quantification was performed by counting number of cells per field of view for 5 images per organ/mouse, the mean of 5 raw counts was calculated and represents one data point per graph.

### Immunohistochemical analysis

Immunohistochemistry was performed on 4.5 µm sections. Sections were de-paraffinized and rehydrated by passing through xylene and a series of ethanols to water. Antigen retrieval was performed by boiling in 10 mM citrate buffer (pH 6.0) for 10 minutes. Endogenous peroxidase activity was blocked with 0.3% hydrogen peroxide for 30 minutes. Sections were then subject to rabbit VECTASTAIN Elite ABC horseradish peroxidase kit following the manufacturers protocols. Sections were developed in DAB for 5 minutes, counterstained in hematoxylin, dehydrated with ethanol, cleared in xylene and mounted in DPX. Primary antibodies; Anti-Ki67 (Thermo Scientific, Fremont, CA, USA; clone: SP6; 1:200), Anti-p-H3 (Merck Millipore, Germany; 06–570: Anti-phospho-Histone H3 (Ser10) Antibody; 1:500), Anti-lysozyme (Life Technologies; A0099), Anti- Chromogranin A (Abcam; ab15160), Anti-CD11b antibody (Abcam; ab133357; 1:4000), Anti-CD3 (Themo Scientific, RM-9107-R7; prediluted), Anti-CD45R (Thermo Scientific, RA3-6B2, 1:100). Alcian blue staining was performed following standard procedures in 1% Alcian blue in 3% acetic acid, pH 2.5.

### Tissue regeneration studies

Liver damage was induced in female and male mice (age 8–12 weeks) by a single intraperitoneal injection of CCl_4_ (0.5 ml/Kg in corn oil). Livers were collected 3 days following the CCl_4_ injections and proliferative hepatocytes were quantified. Intestinal damage was induced by 14 Gy of gamma irradiation from a Cs^137^ source and small intestine collected 3 days later.

### Data Availability

All data generated or analysed during this study are included in this published article (and its Supplementary Information files).

## Electronic supplementary material

Supplementary Figures
