# Supplementary material for: Determination of the physiological and pathological roles of E2F3 in adult tissues
Source: Sci Rep. 2017 Aug 30;7:9932. doi: 10.1038/s41598-017-09494-6 (PMC5577339; doi:10.1038/s41598-017-09494-6)
Supplement: Supplementary file 1 — Supplementary Figures [file 41598_2017_9494_MOESM1_ESM.pdf]

Determination of the physiological and pathological roles of E2F3 in adult tissues

Ivonne Gamper, Deborah L. Burkhart, Megan J. Bywater, Daniel Garcia, Catherine H. Wilson, Peter A. Kreuzaler, Mark J. Arends, Yao-Wu Zheng, Alessandra Perfetto, Trevor D. Littlewood, and Gerard I. Evan

Supplementary Figures

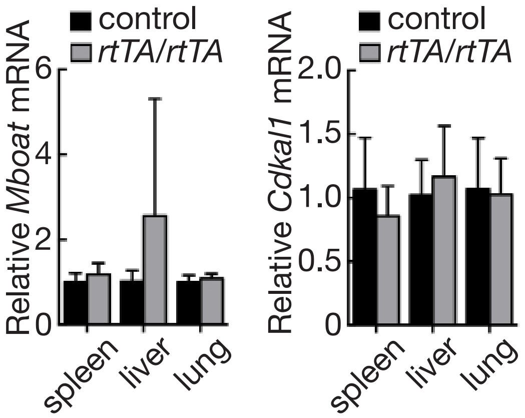

Supplementary Fig. 1| Expression of genes proximal to *E2f3* are unaffected by rtTA induced expression of *E2f3*.

Quantitative RT-PCR analysis of *Mboat* and *Cdkal1* in adult control (*n*=5 (spleen,lung), 4 (liver)) and $E2f3^{TRE/TRE}$;$Rosa26^{rtTA/rtTA}$ (*n*=3) mouse tissues. Expression is normalized to *Actin* and relative to the mean of the control samples within each tissue. Error bars, s.e.m.

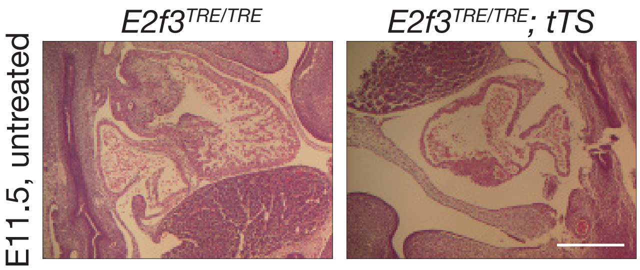

Supplementary Fig. 2| Repression of *E2f3* in $E2f3^{TRE/TRE}$;*β-actin-tTS* embryos results in reduced heart trabeculation.

Haematoxylin and eosin stained embryo hearts from $E2f3^{TRE/TRE}$;*β-actin-tTS* and $E2f3^{TRE/TRE}$ control 11.5 d.p.f. embryos developed in the absence of tetracycline.

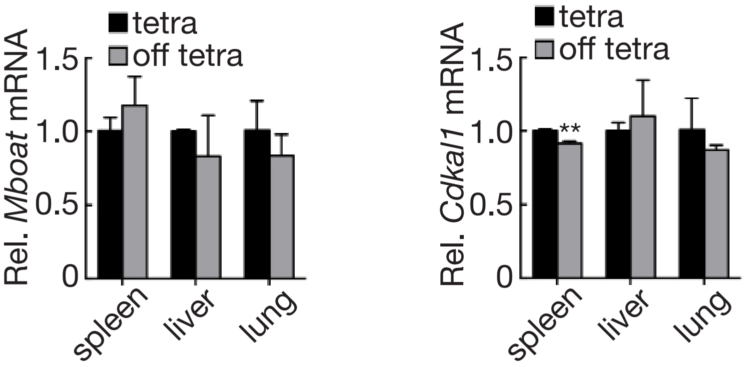

Supplementary Fig. 3| Expression of genes proximal to *E2f3* are unaffected by tTS-dependent repression of *E2f3*.
Quantitative RT-PCR analysis of *Mboat* and *Cdkal1* expression in *E2f3$^{TRE/TRE}$;β-actin-tTS* mouse tissues in the presence (n=2), or post removal of tetracycline for ≥1 week (n=3 (spleen, lung), 4 (liver)). Expression is normalized to *Actin* and relative to the mean of the control samples within each tissue. Error bars, s.e.m. Two-tailed t-test: tetra vs off tetra; *P*=0.009** (*Cdkal1*, spleen).

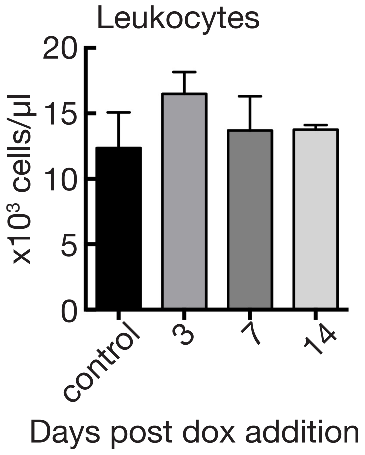

Supplementary Fig. 4| Peripheral leukocyte numbers trend towards a transient increase following rtTA induced expression of *E2f3*.
Leukocyte counts on peripheral blood isolated from control (*n*=2 three days post dox, *n*=2 seven days post dox) and *E2f3$^{TRE/TRE}$;Rosa26$^{rtTA/rtTA}$* (*n*=3, three and seven days post dox; *n*=2, 14 days post dox) mice at the indicated number of days post addition of Doxcycline to the drinking water. Error bars, s.e.m. Two-tailed t-test: control vs 3 days; *P*=0.071.

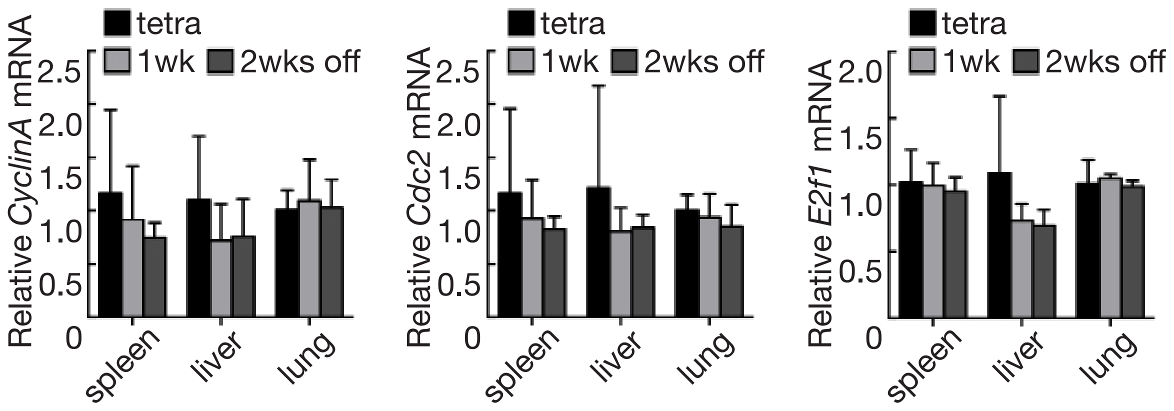

Supplementary Fig. 5| E2F target genes are unaffected by repression of *E2f3* in adult mouse tissues.
Quantitative RT-PCR analysis of *E2f1, Cdc2* and *CyclinA* expression in adult *E2f3$^{TRE/TRE}$;β-actin-tTS* mouse tissues maintained on tetracycline (tetra, *n*=3) and after removal from tetracycline for the times indicated (1wk, *n*=4; 2wks off, *n*=3). Expression is normalized to *HPRT* and relative to the mean of the control samples within each tissue. Error bars, s.e.m.

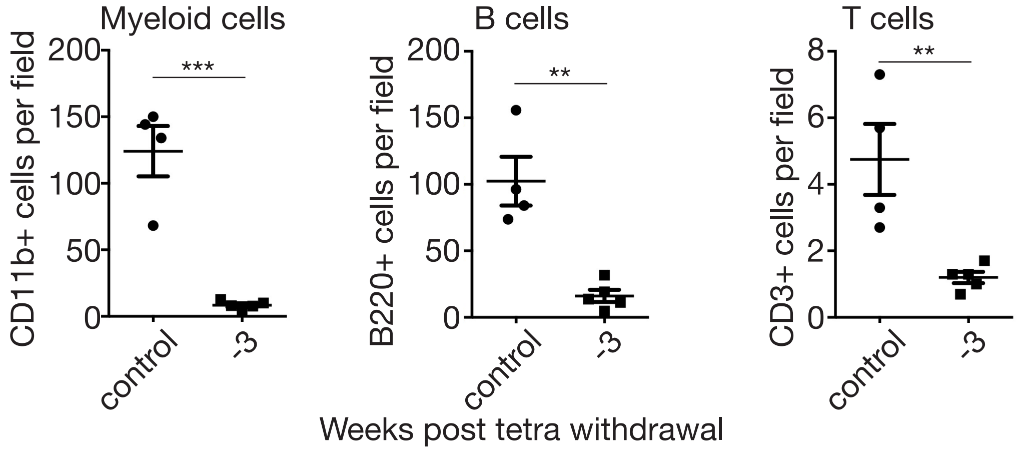

Supplementary Fig. 6| Repression of activator *E2fs* results in attrition of leukocytes in the bone marrow. Histogram of average numbers of CD11b (myeloid), B220 (B-cells) and CD3 (T-cells) positive cells from three fields of view in the bone marrow of *E2f1$^{-/-}$;E2f2$^{-/-}$;E2f3$^{TRE/TRE}$* control (n=4) and *E2f1$^{-/-}$;E2f2$^{-/-}$;E2f3$^{TRE/TRE}$;β-actin-tTS* mice following withdrawal of 100 mg/L tetracycline for three weeks (-3, n=5). Two-tailed t-test: *P*=0.0002*** (myeloid), *P*=0.0014** (B cells), *P*=0.0076** (T cells).

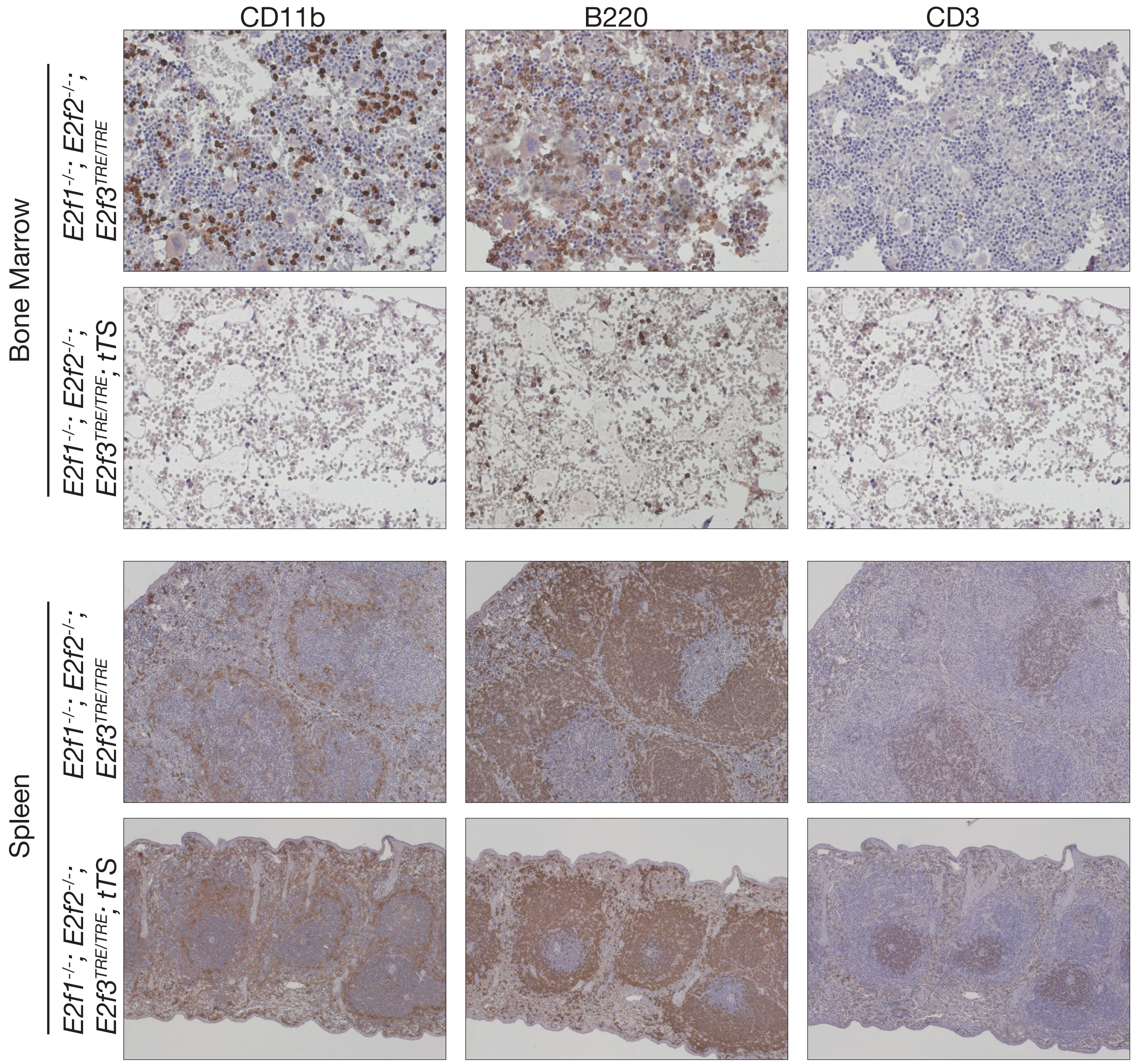

Supplementary Fig. 7| Repression of activator *E2fs* results in attrition of leukocytes in the bone marrow and spleen. CD11b, B220 and CD3 stained images of the bone marrow and spleen for the indentification of myeloid, B and T cells respectively in *E2f1$^{-/-}$;E2f2$^{-/-}$;E2f3$^{TRE/TRE}$* control and *E2f1$^{-/-}$; E2f2$^{-/-}$;E2f3$^{TRE/TRE}$;β-actin-tTS* mice 3 weeks after withdrawal of tetracycline. Representative images from n≥4 mice.

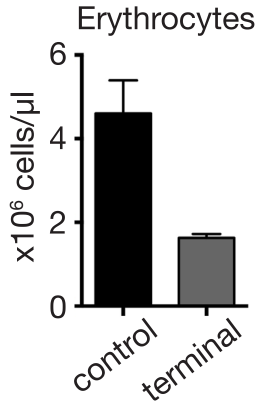

Supplementary Fig. 8| Repression of activator *E2fs* results in erythrocytopenia.
Histogram of total numbers of erythrocytes in *E2f1$^{-/-}$;E2f2$^{-/-}$;E2f3$^{TRE/TRE}$* control (n=8) and *E2f1$^{-/-}$;E2f2$^{-/-}$;E2f3$^{TRE/TRE}$;β-actin-tTS* mice following withdrawal of 100 mg/L tetracycline until mice presented as moribund (terminal, n=2).

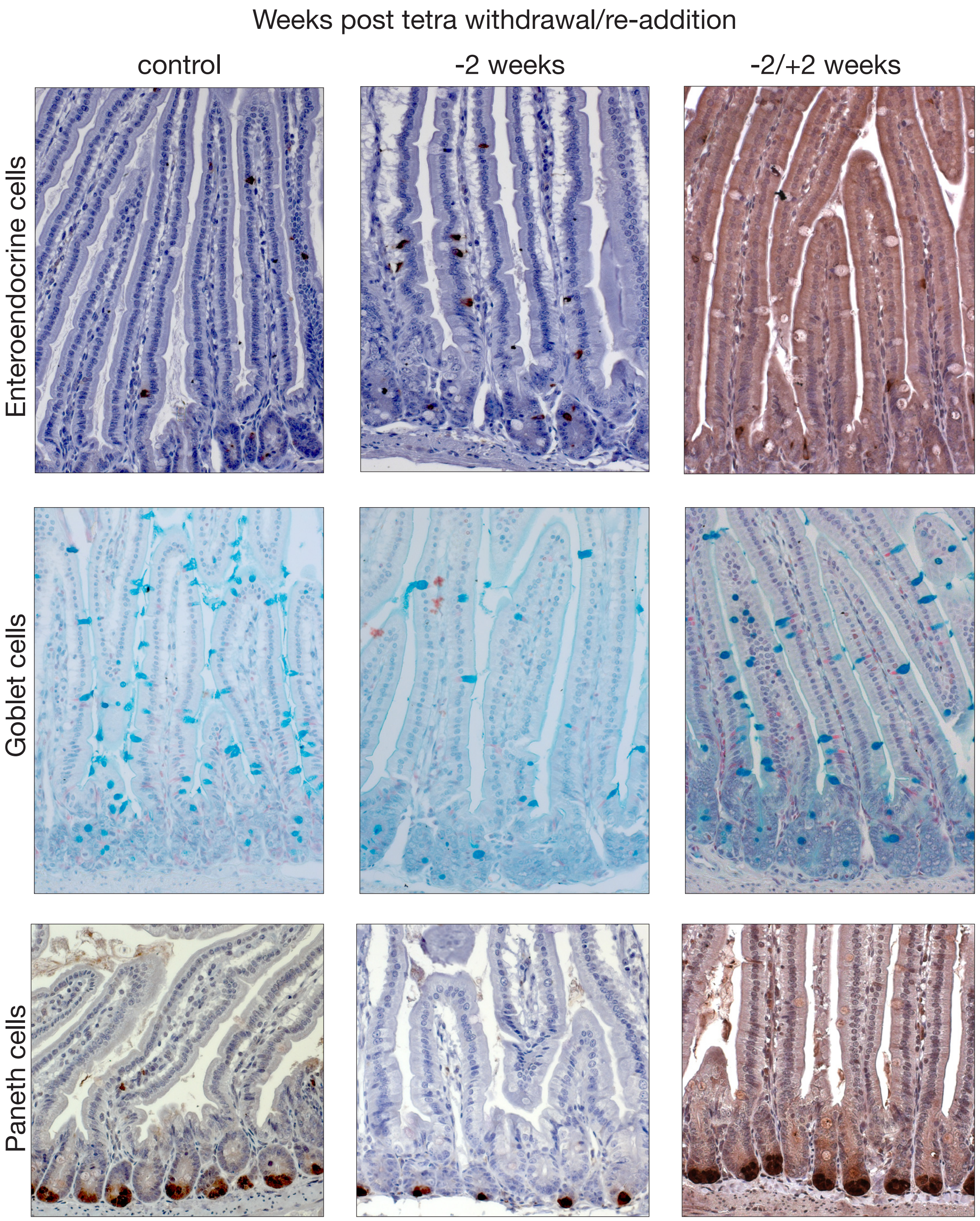

Supplementary Fig. 9| Repression of *E2f3* in the absence of *E2f1* and *E2f2* results in altered proportions of differentiated cell types in the intestinal epithelium that can be rescued with the restoration of *E2f3* expression.

Chromogranin A, Alcian blue and lysozyme stained images of the intestinal epitelium for the in-dentification of Enteroendocrine, Goblet and Paneth cells respectively in *E2f1*$^{-/-}$;*E2f2*$^{-/-}$;*E2f3*$^{TRE/TRE}$ control and *E2f1*$^{-/-}$; *E2f2*$^{-/-}$;*E2f3*$^{TRE/TRE}$;*β-actin-tTS* mice 2 weeks after withdrawal of tetracycline and 2 weeks post re-addition. Representative images from n≥2 mice.

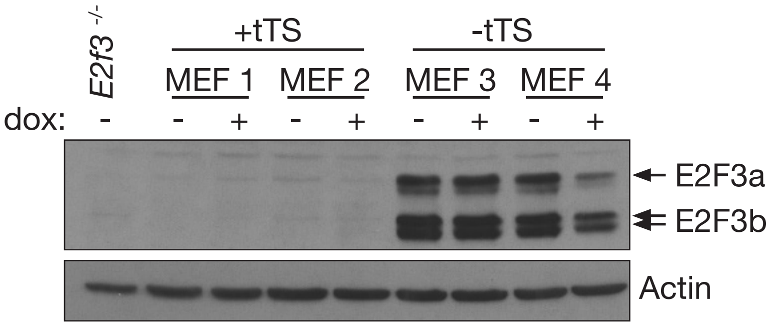

Supplementary Fig. 10| Repression of *E2f3* is irreversible when tTS is active during embryonic development.

Western blot analysis of E2F3 protein levels in *E2f3*$^{TRE/TRE}$ (-tTS) and *E2f3*$^{TRE/TRE}$*; βactin-tTS* (+tTS) cultured mouse embryonic fibroblasts (MEFs), generated in the absence of doxycycline. MEFs were then grown in culture with (+) or without doxycycline (-), for at least two passages, as indicated. Replicate samples represent cultures derived from independently isolated embryos. Expression of Actin is included as a loading control.

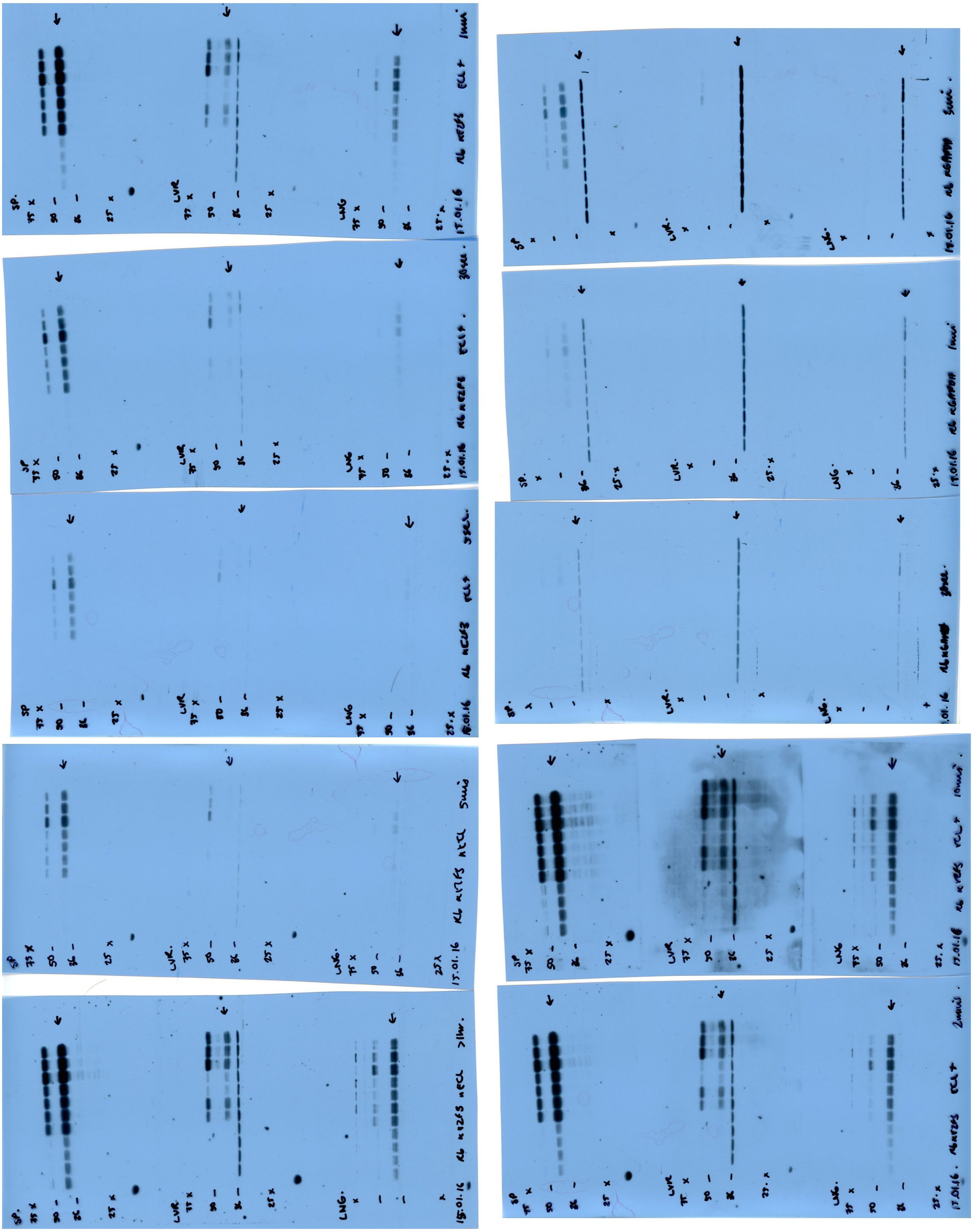

Supplementary Fig. 11| Uncropped images of blots represented in Figure 2c.
Multiple exposures also included.

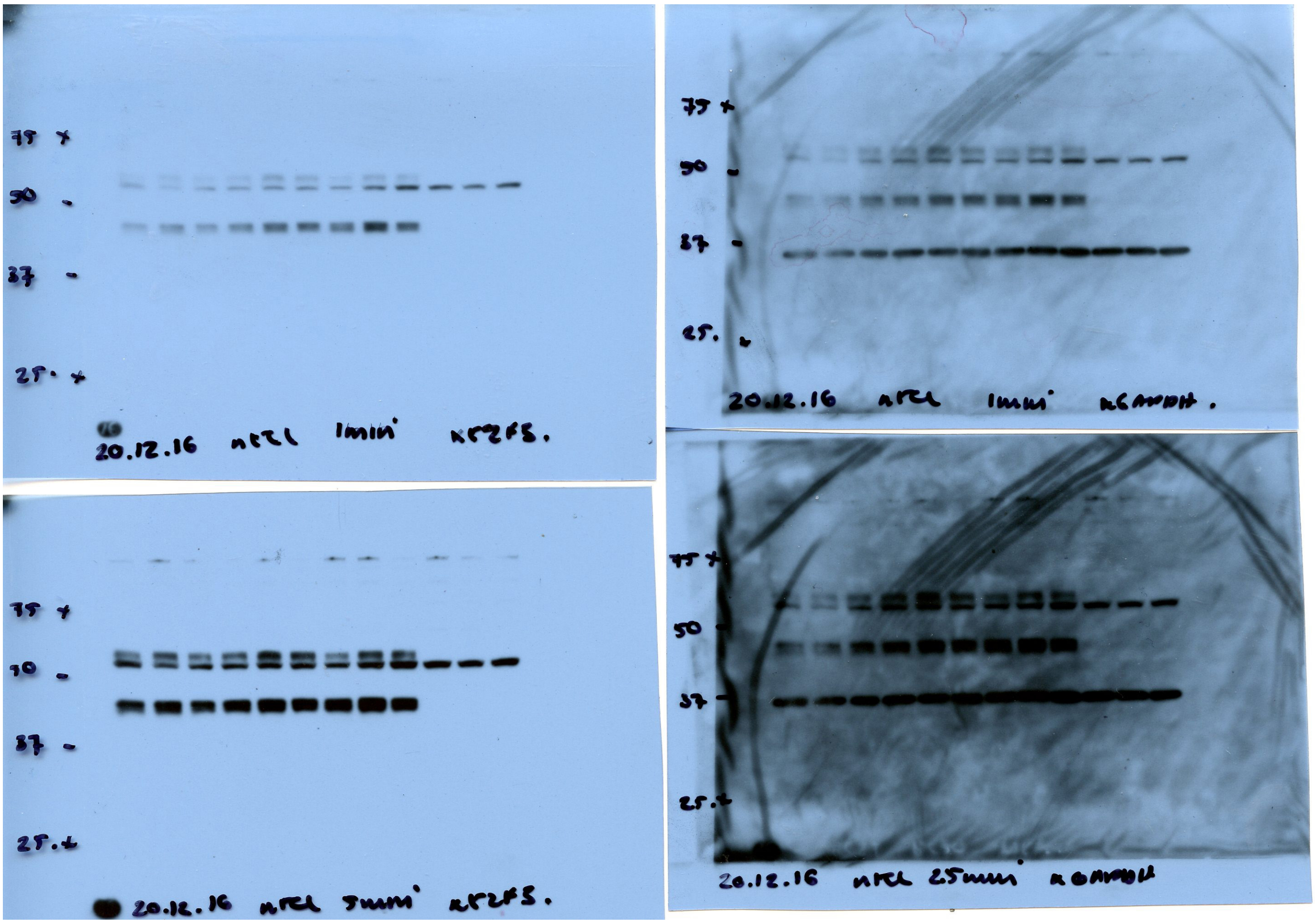

Supplementary Fig. 12| Uncropped images of blots represented in Figure 3c.
Multiple exposures also included.

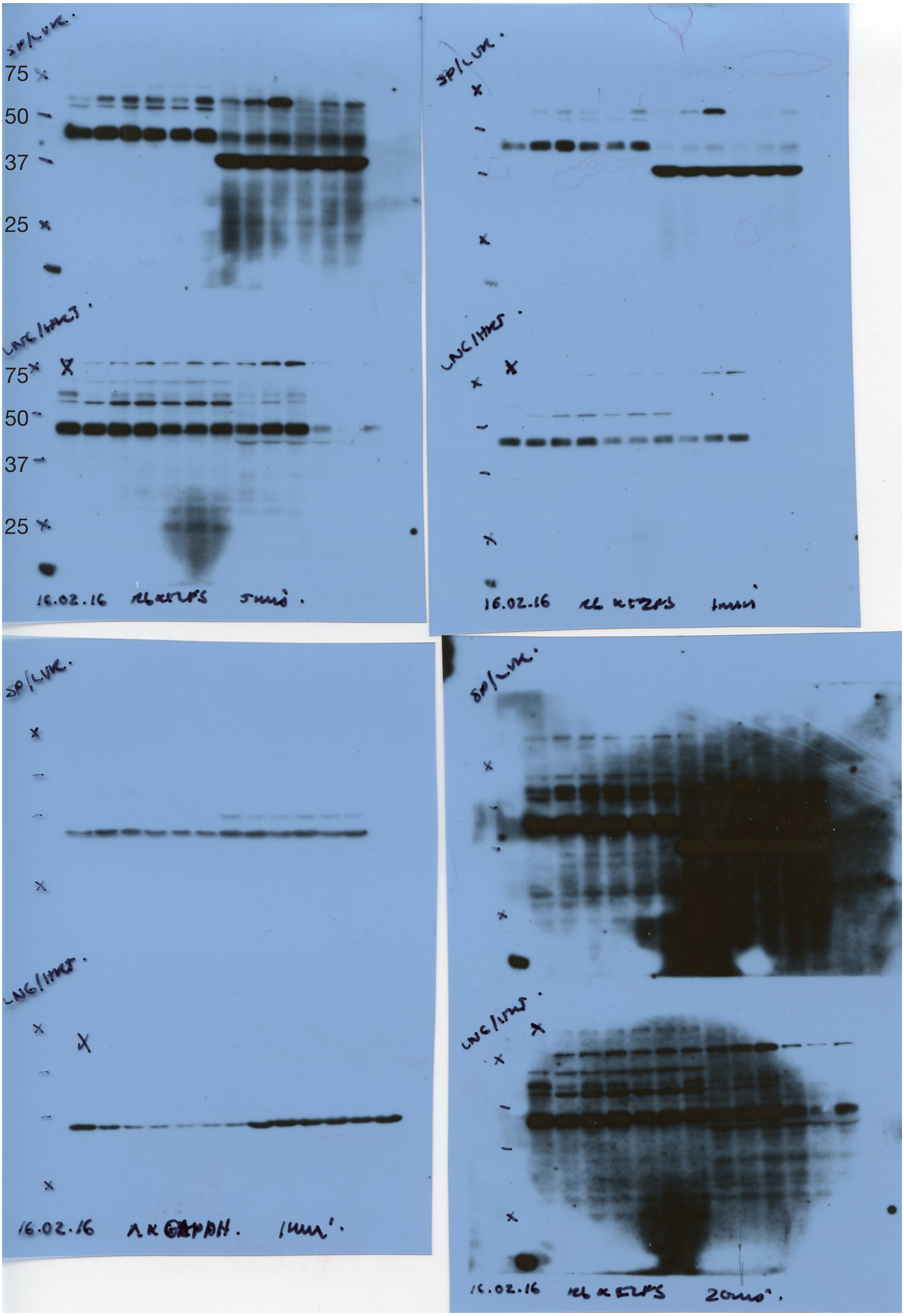

Supplementary Fig. 13| Uncropped images of blots represented in Figure 3d.
Multiple exposures also included. “X” on the first sample on LNG blot denotes a mis-loaded sample.

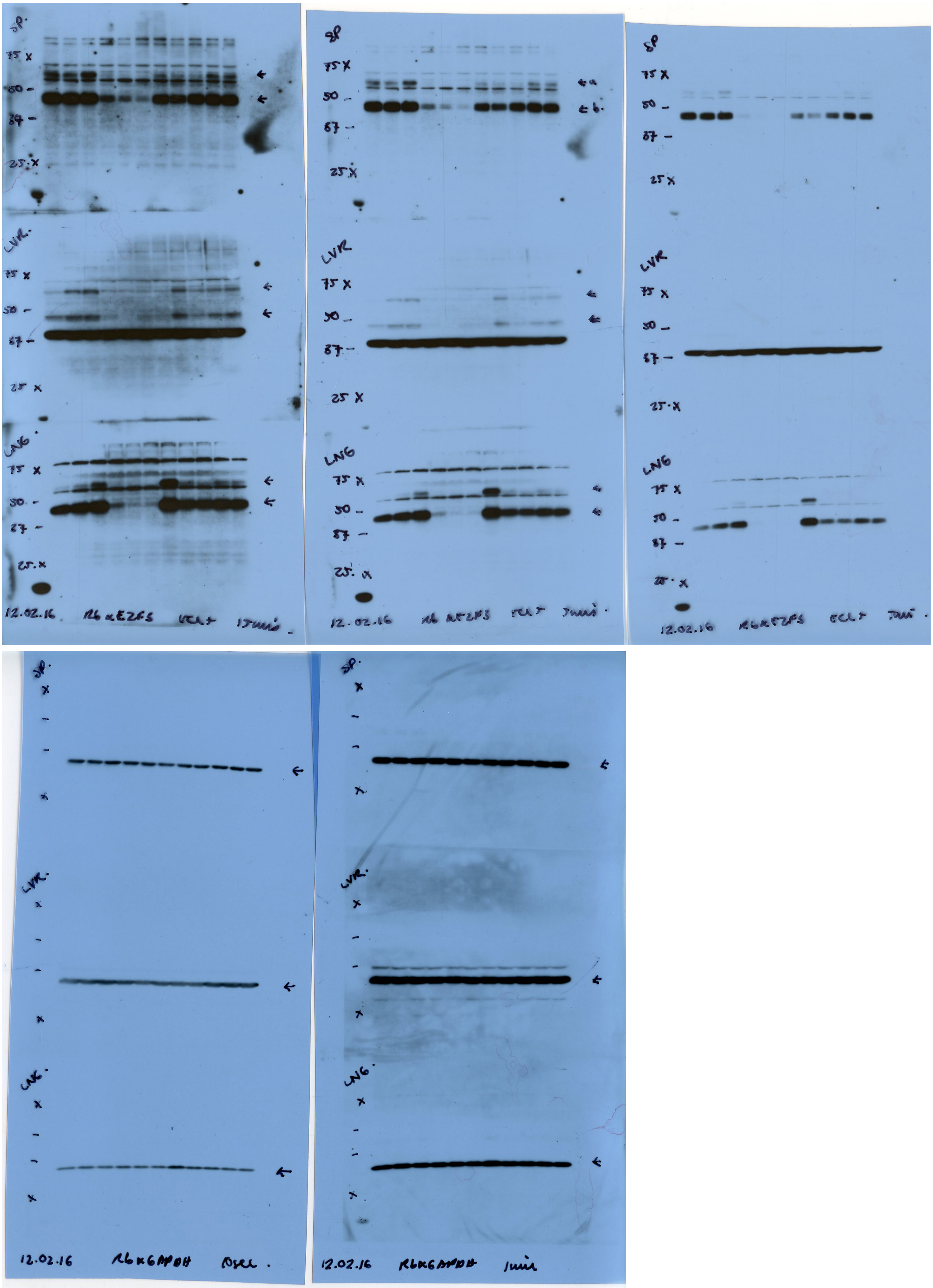

Supplementary Fig. 14| Uncropped images of blots represented in Figure 3f.
Multiple exposures also included.
